# Supplementary material for: A data-driven interactome of synergistic genes improves network-based cancer outcome prediction
Source: PLoS Comput Biol. 2019 Feb 6;15(2):e1006657. doi: 10.1371/journal.pcbi.1006657 (PMC6380593; doi:10.1371/journal.pcbi.1006657)
Supplement: S1 Text — (DOCX) [file pcbi.1006657.s001.docx]

Supplementary Material

# S1 text

## Gene expression preprocessing

For the METABRIC dataset, clinical data was collected from the Synapse Commons archive (syn2133322; www.synapse.org) and normalised gene expression profiles were retrieved from the European genome-phenome archive (EGAS00000000083). For this study, gene expression was measured using Illumina HT-12 v3 platform. For the ACES dataset (see **S1 Table** for accession number of individual studies in ACES), apart from quantile normalization and batch effect removal, no preprocessing was performed. TCGA breast invasive carcinoma (BRCA) gene expression profiles were retrieved from UCSC Xena Browser [(1)](https://paperpile.com/c/s9jEPr/YQfjL). These data were obtained using Agilent 244K custom gene expression (G4502A_07_3) microarrays.


## Network preprocessing

The Human Interactome (HumanInt, vII-14) network [(2)](https://paperpile.com/c/s9jEPr/0xGA0) is collected from interactome.baderlab.org. This network does not have weighted links and hence all interactions are utilized (n=14057). BioPlex v2.0 [(3)](https://paperpile.com/c/s9jEPr/ipl63) is obtained from bioplex.hms.harvard.edu. The weights for each pair of genes is collected from the p(Interaction) column which reflects the likelihood of an interaction to be a true positive. The organism specific version of BioGRID (Homo sapiens, v3.4.155) [(4)](https://paperpile.com/c/s9jEPr/ujy0k) was obtained from thebiogrid.org and the “score” column is used for link weights. The Homo Sapiens version of the STRING network (9606, v10) [(5)](https://paperpile.com/c/s9jEPr/t4Qxz) is collected from string-db.org and the “combined score” is utilized for link weights. The tissue specific networks are downloaded from the HumanBase [(6)](https://paperpile.com/c/s9jEPr/mn6Bn) website (hb.flatironinstitute.org). Each link in these networks has a weight which reflects the tissue specificity of that interaction.

UniProt (used by IntAct) and Ensembl gene IDs (used by STRING) are converted to HGNC IDs using Ensembl Biomart [(7)](https://paperpile.com/c/s9jEPr/upBDE). Entrez IDs (used by BioPlex, HumanBase and BioGRID) are mapped to HGNC using the Hugo server (genenames.org). HumanInt uses HGNC IDs to refer to genes and hence no further conversion is needed for this network.

## Lasso and Lasso derivatives

We employ the Lasso (least absolute shrinkage and selection operator), proposed by Tibshirani [(8, 9)](https://paperpile.com/c/s9jEPr/oLhT+5bQ8) which aims to regularize a regression model by by “shrinking” the non-essential regression coefficients _
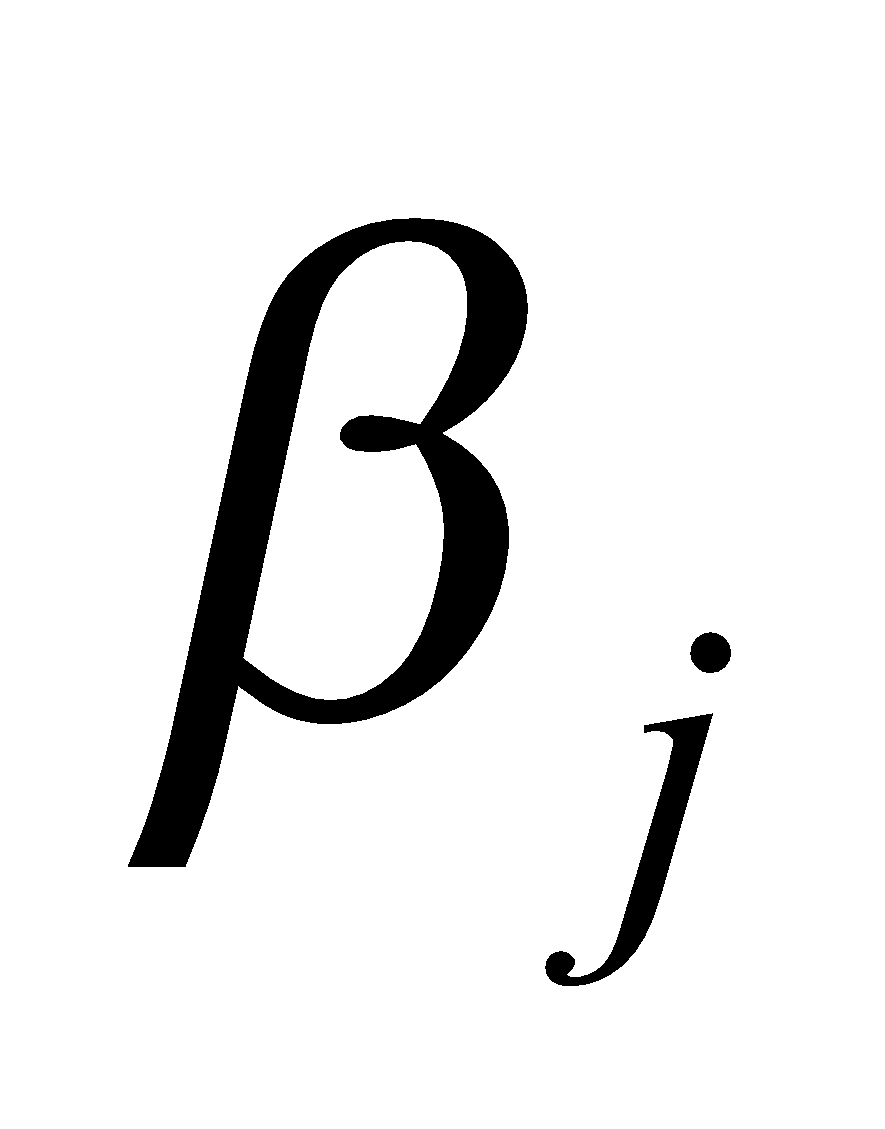
_ to zero while preserving the prediction power (minimal MSE) of the model. The cost function of Lasso is as follows:

_
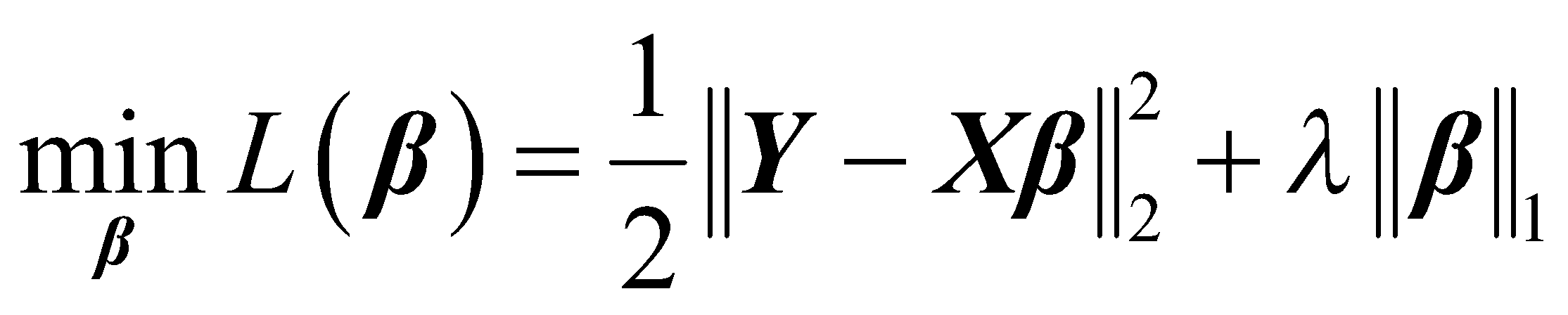
_

_
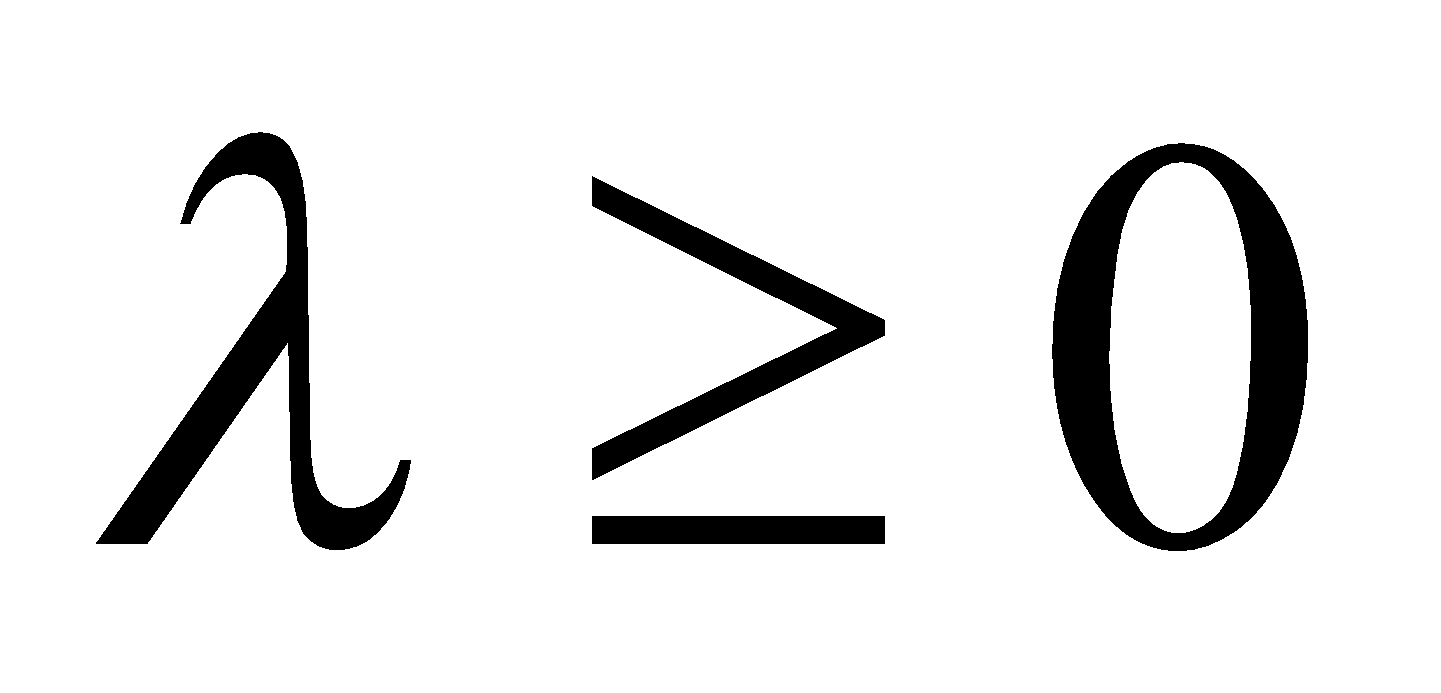
_

Where _
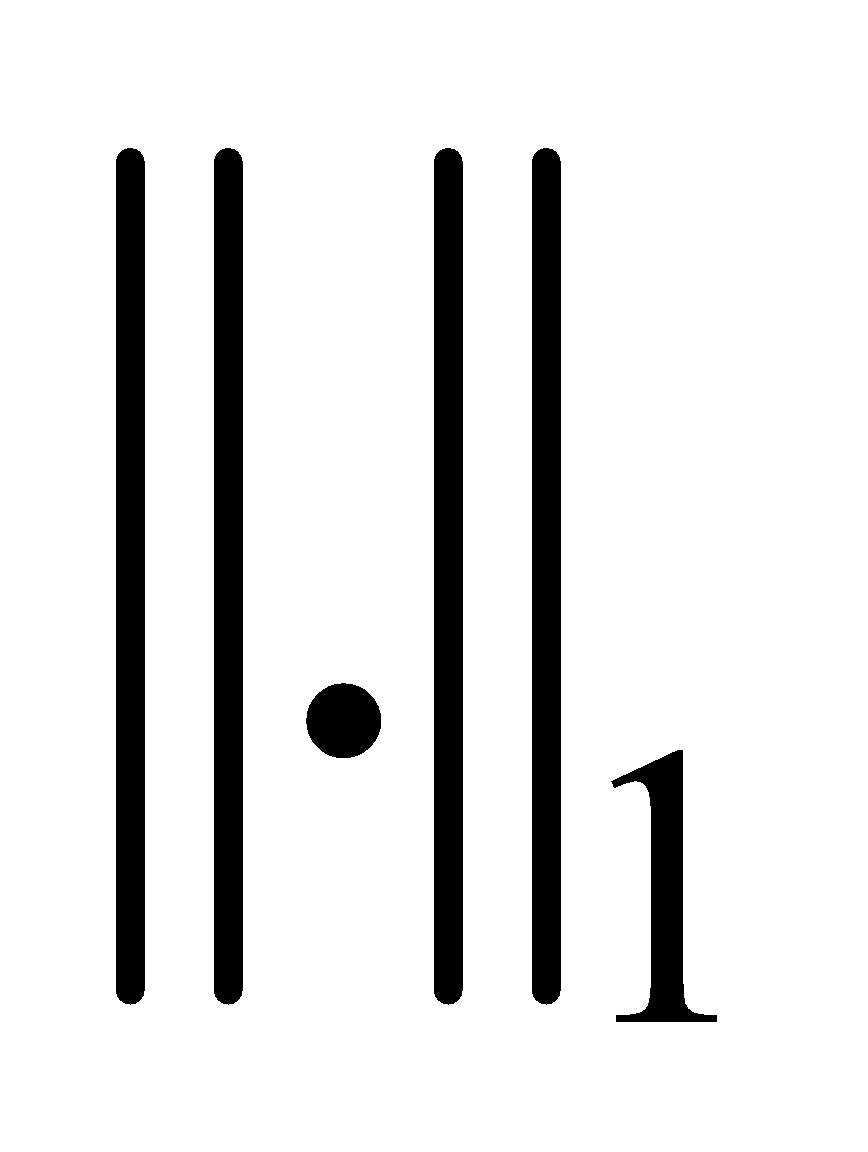
_ is the _
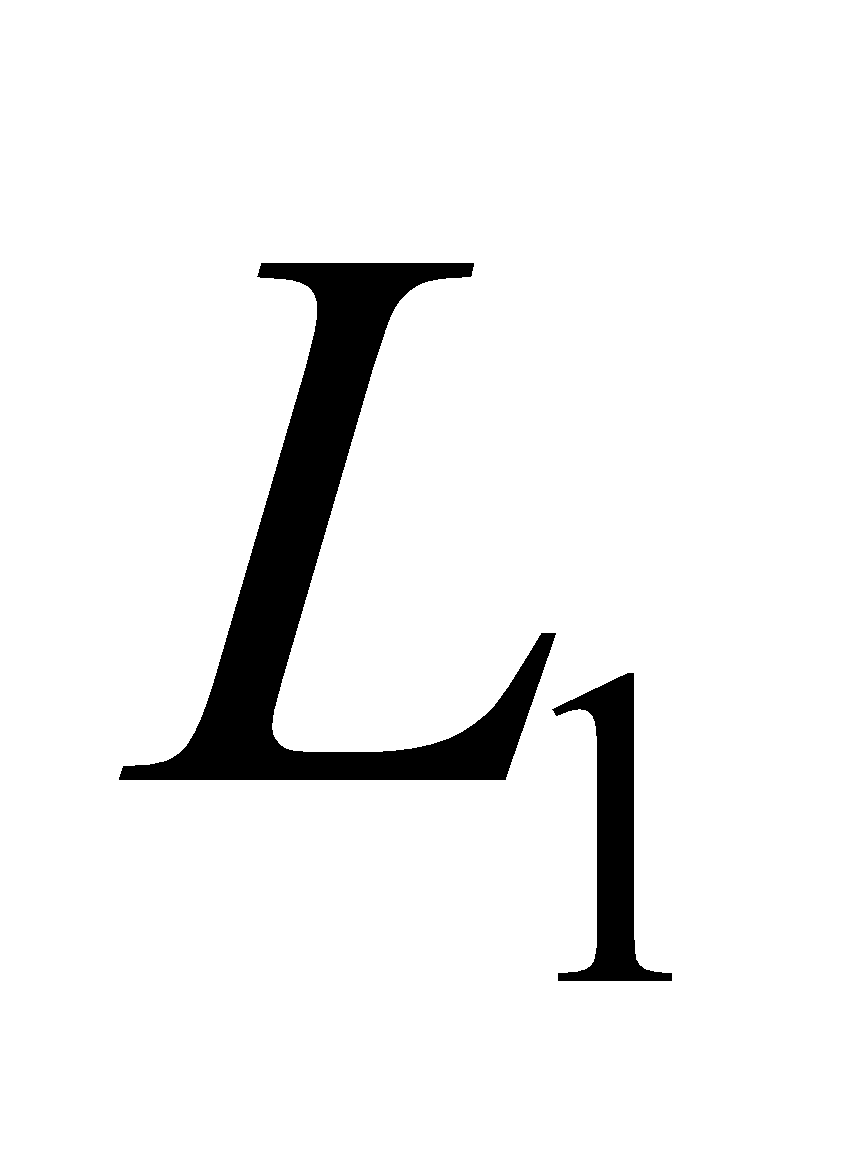
_ norm e.g. _
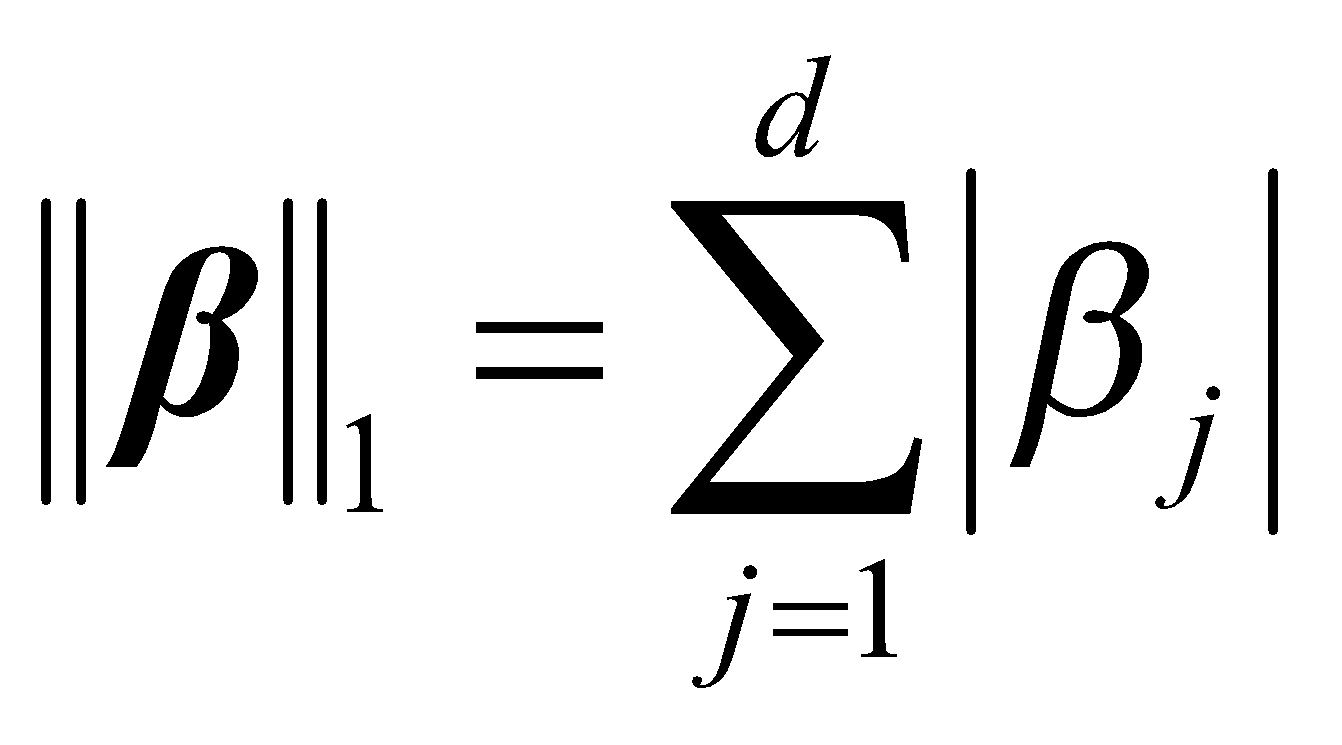
_. The added penalty term encourages sparsity in the weights of the solution vector _
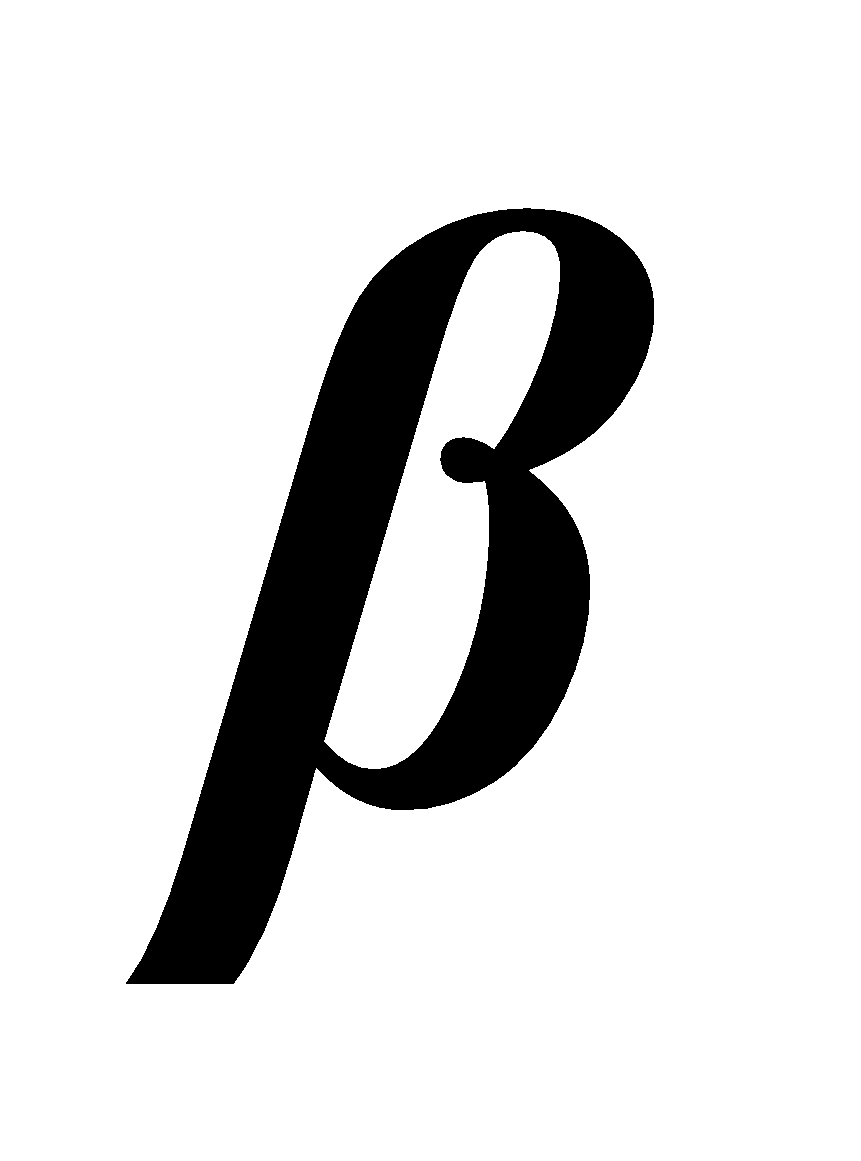
_ and thus automatically leads to feature/model selection. Meanwhile, the scale regularizer _
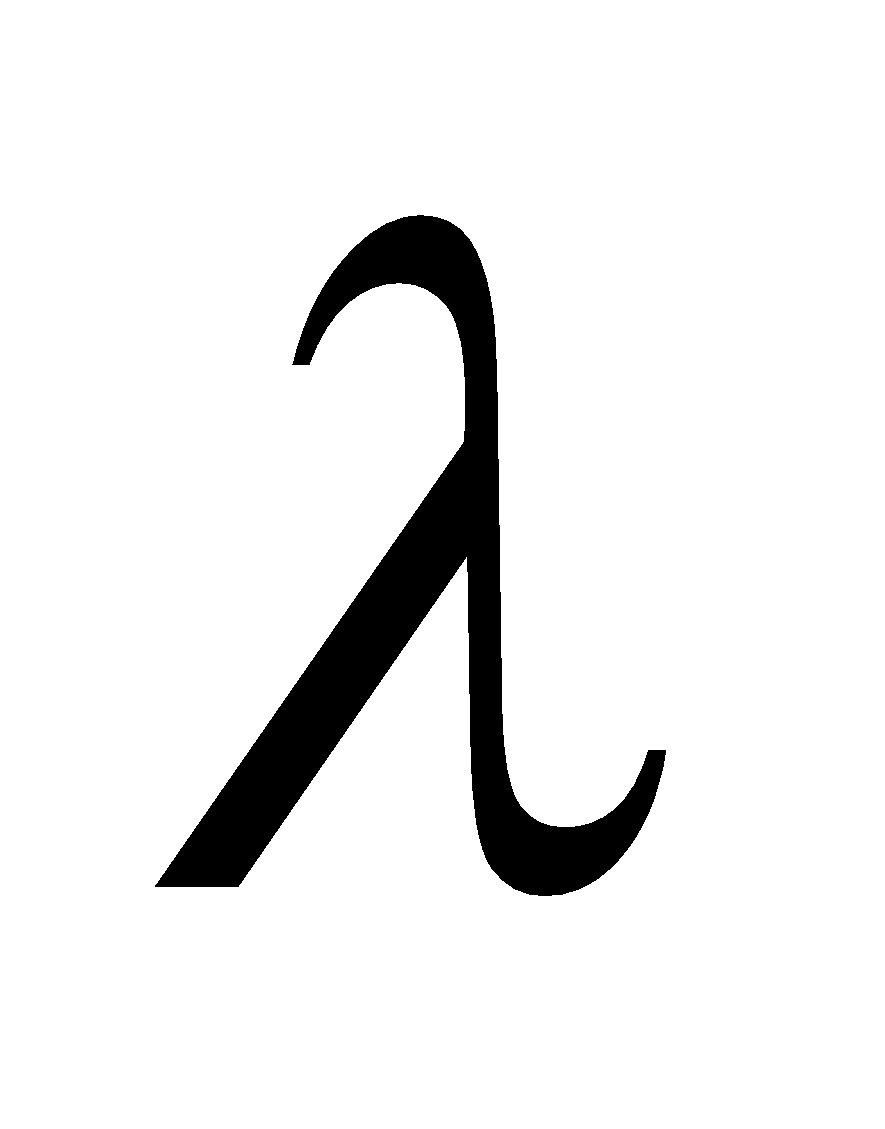
_ provide the balance between MSE (first term of cost function) and weight shrinkage. The value for _
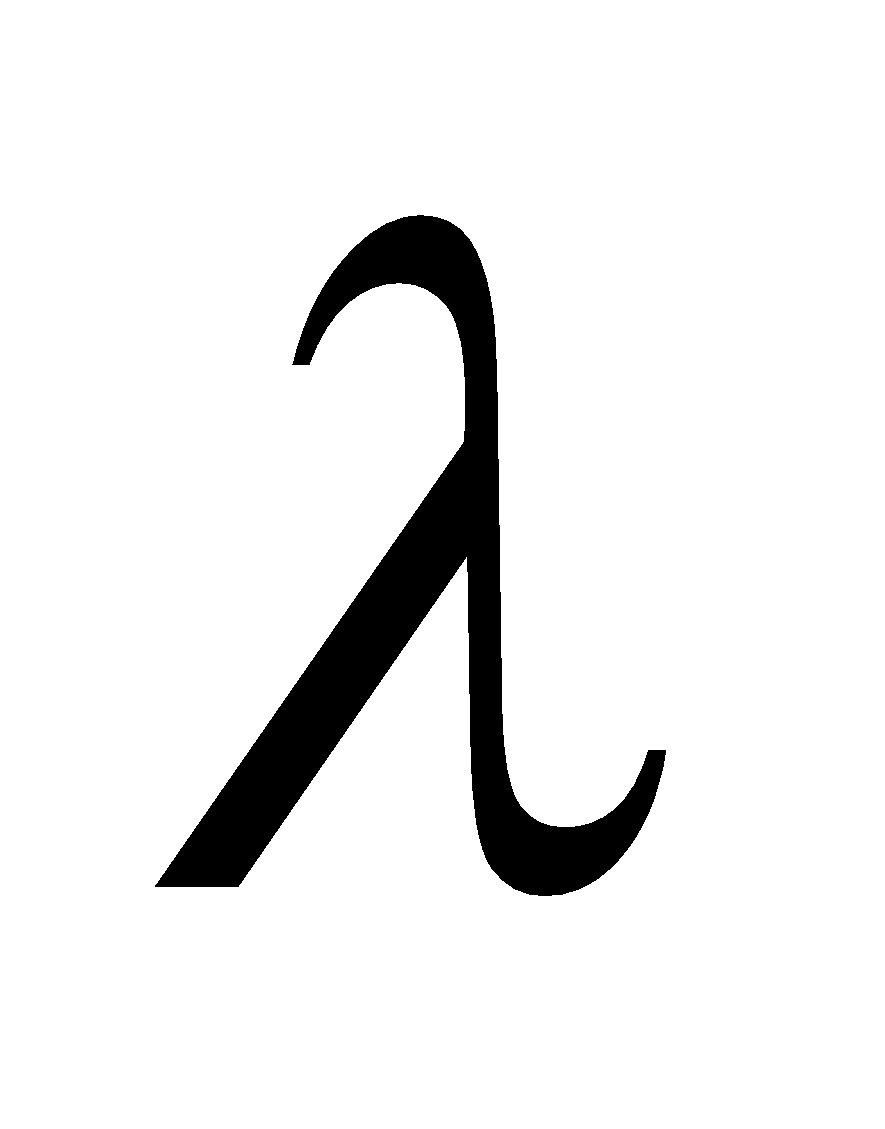
_is typically found using a cross-validated grid search.

Yuan & Lin proposed a new version of Lasso capable of selecting groups of features by solving the following convex optimization problem [(10)](https://paperpile.com/c/s9jEPr/I1BUy):

_
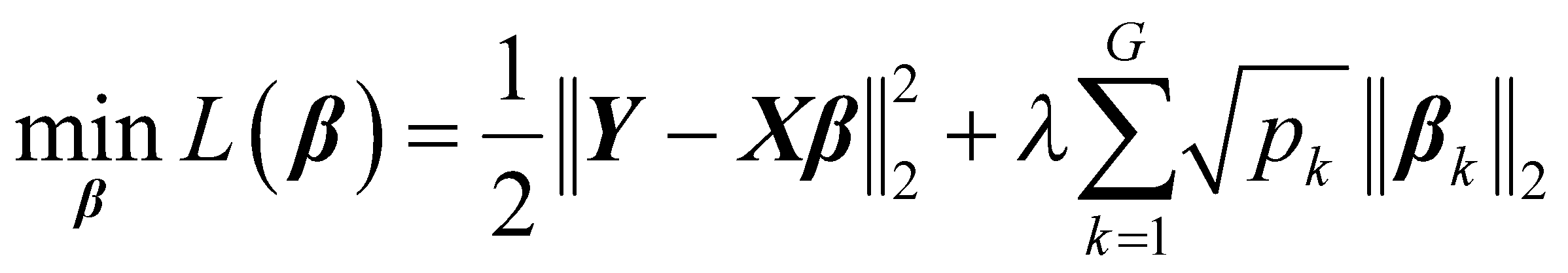
_

_
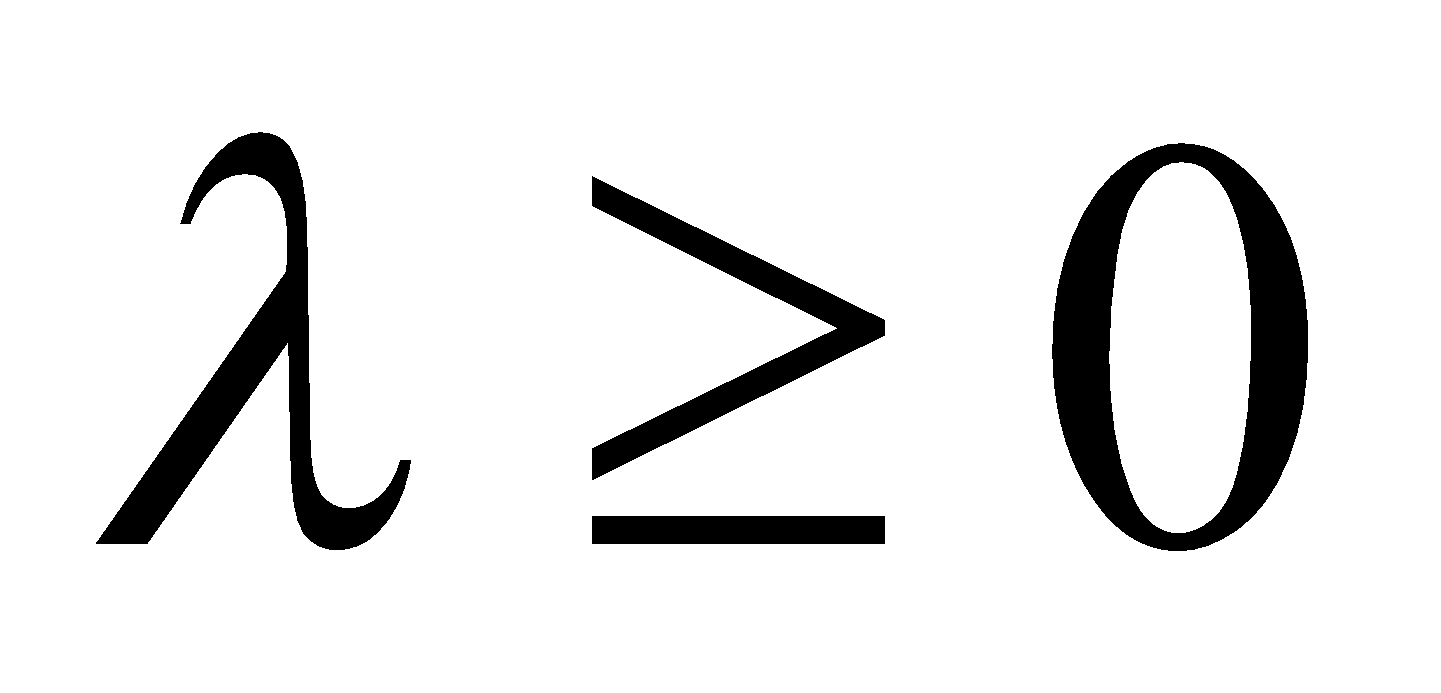
_

Here, the _
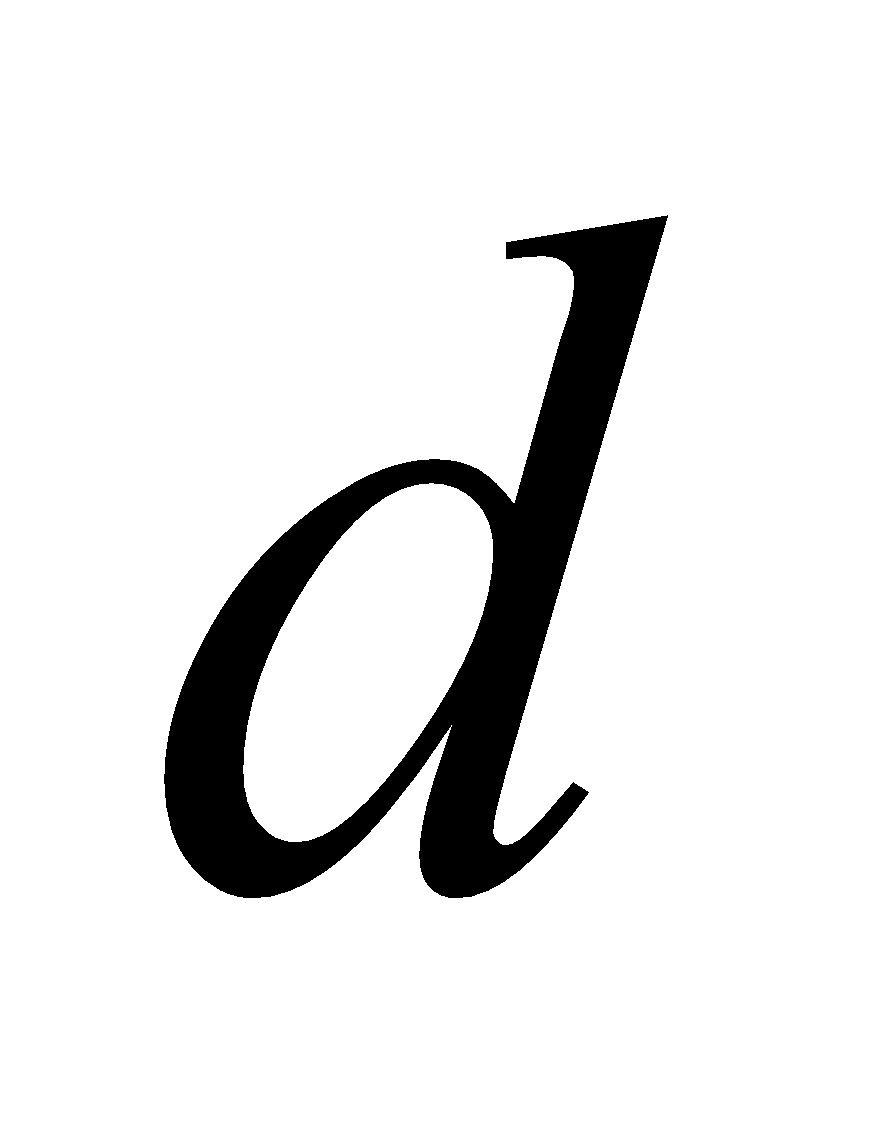
_ predictors are divided into _
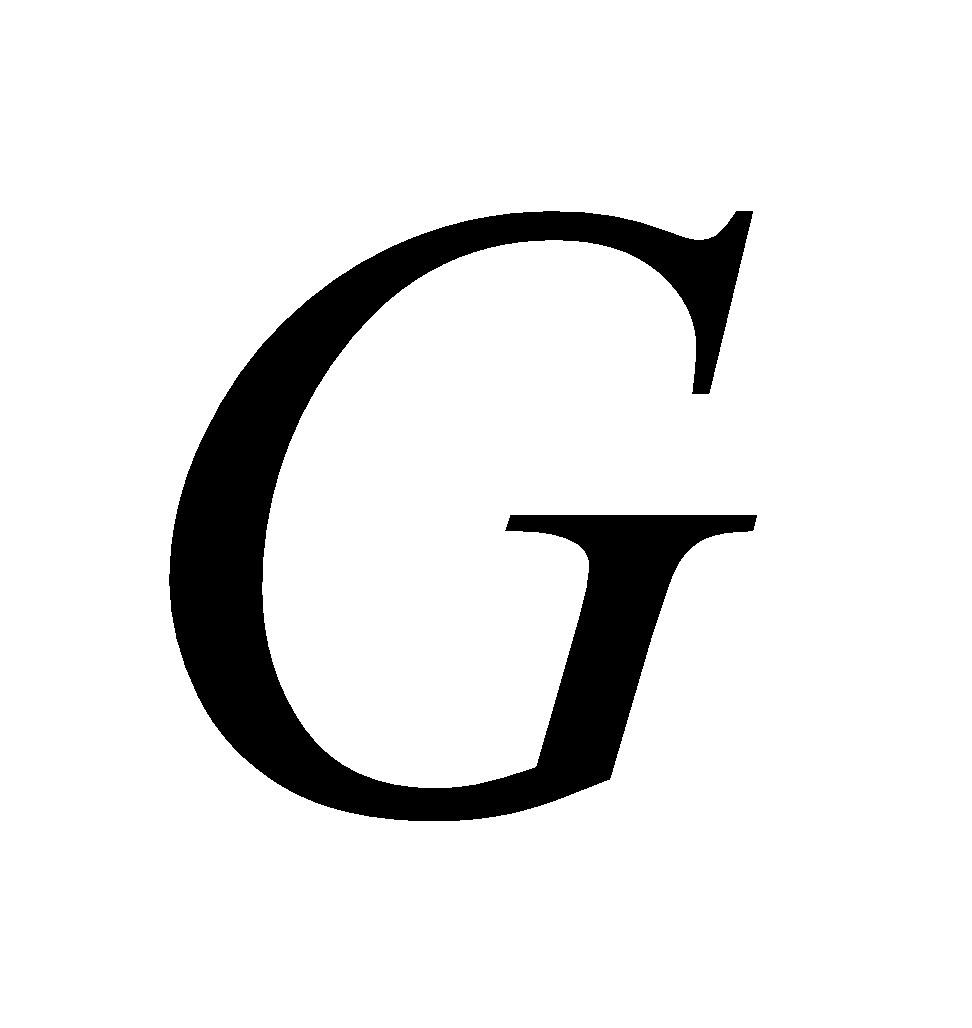
_ groups and _
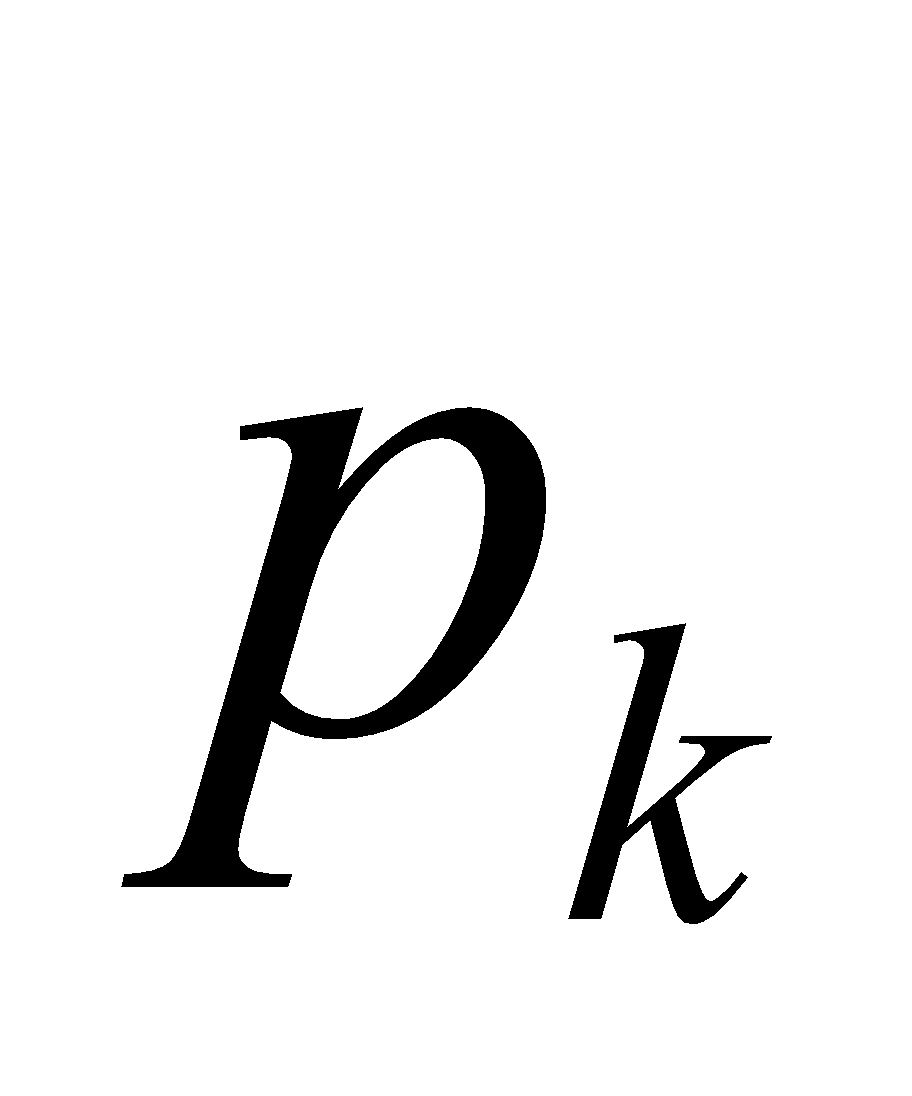
_ where _
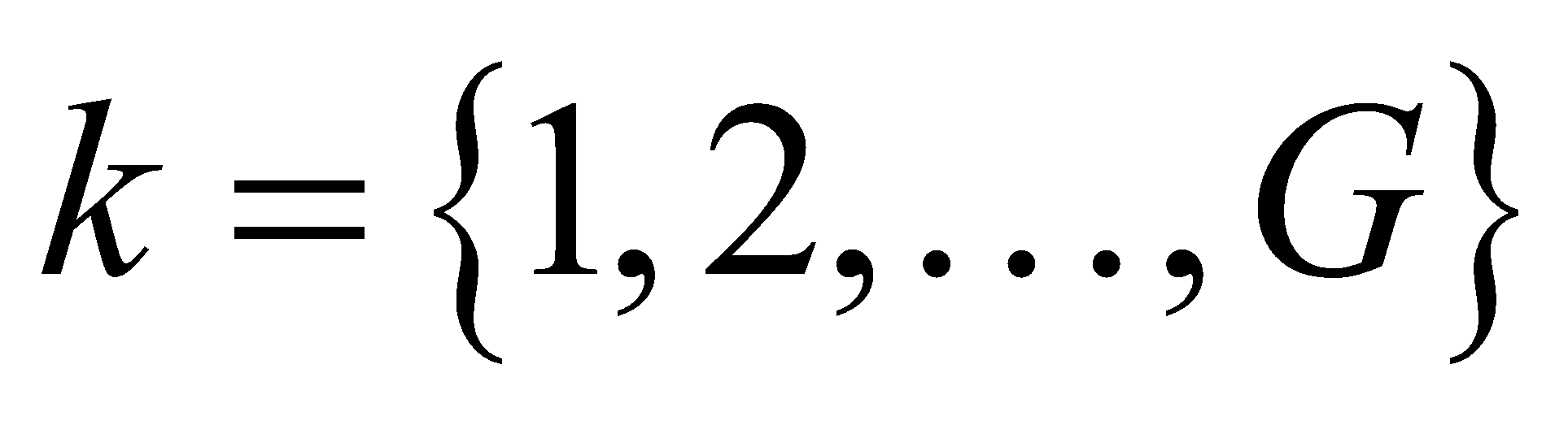
_ determines the number of observations in _
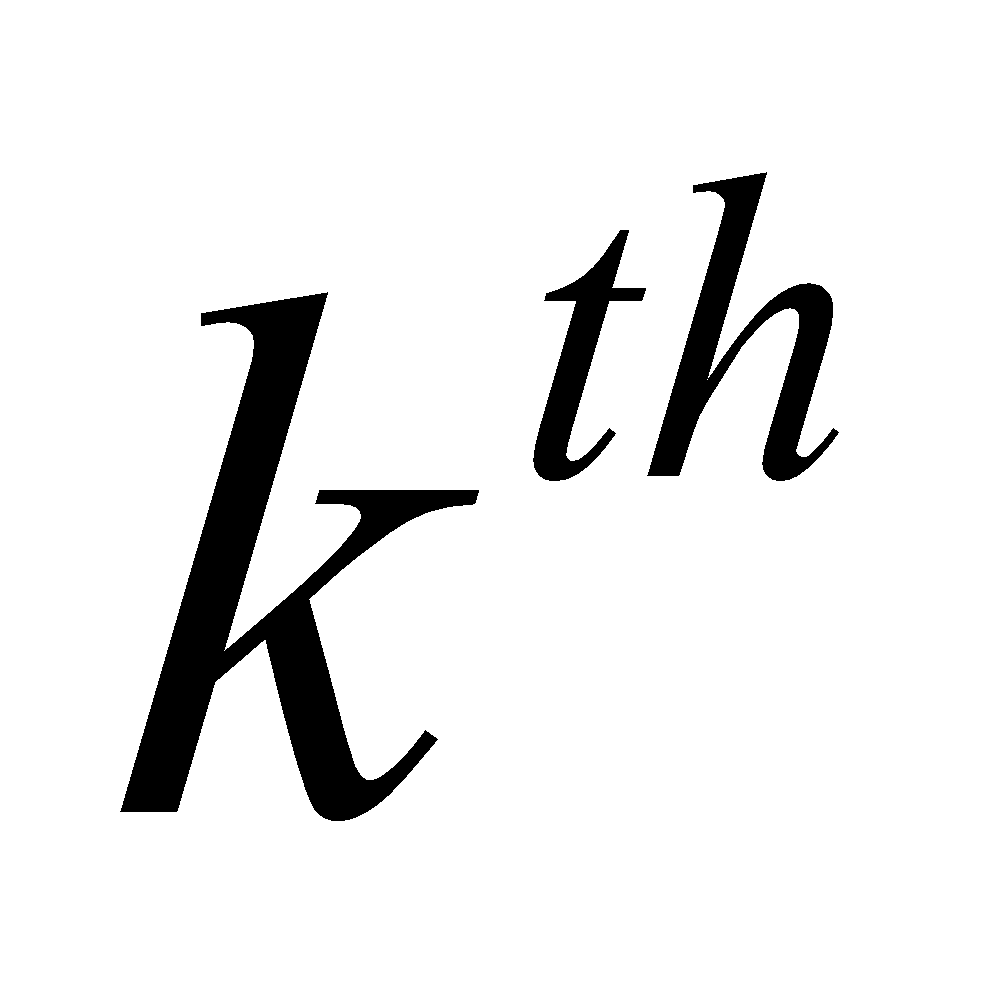
_ group. For ease of notation, we use a matrix _
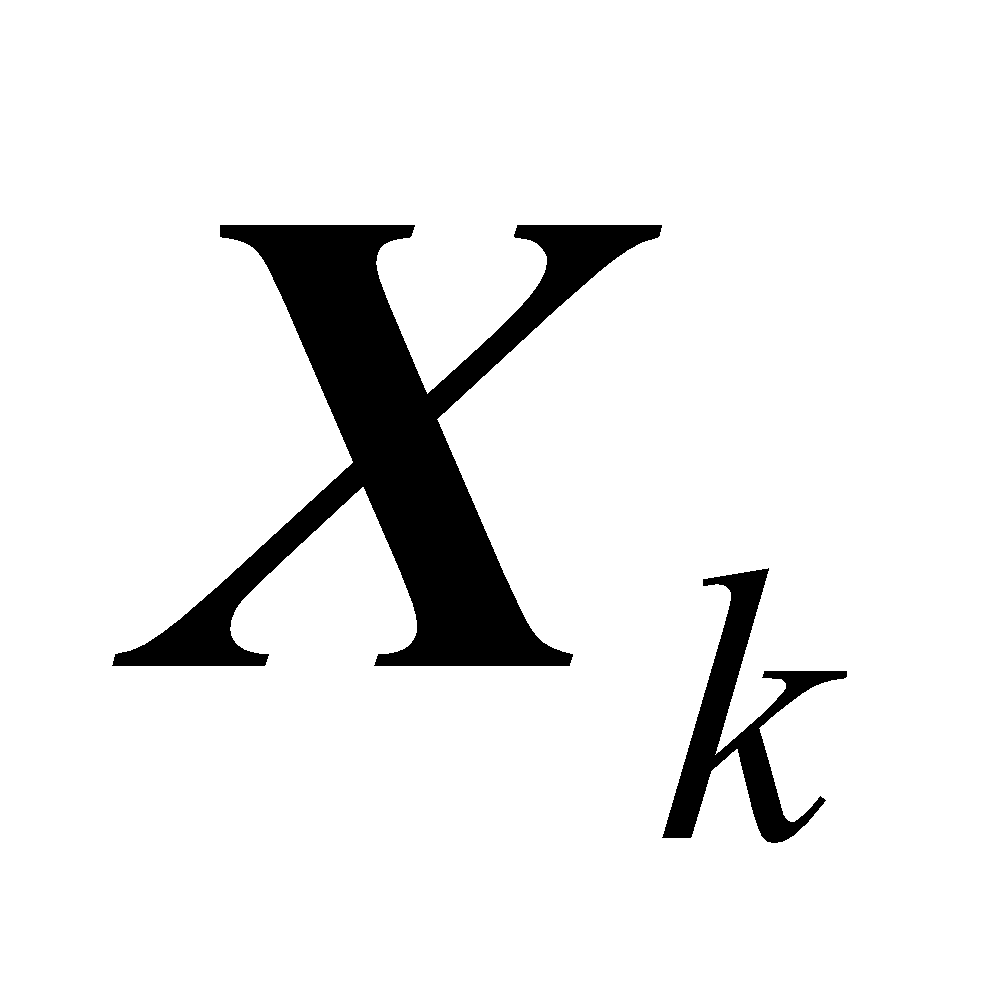
_ to represent the observation corresponding to the _
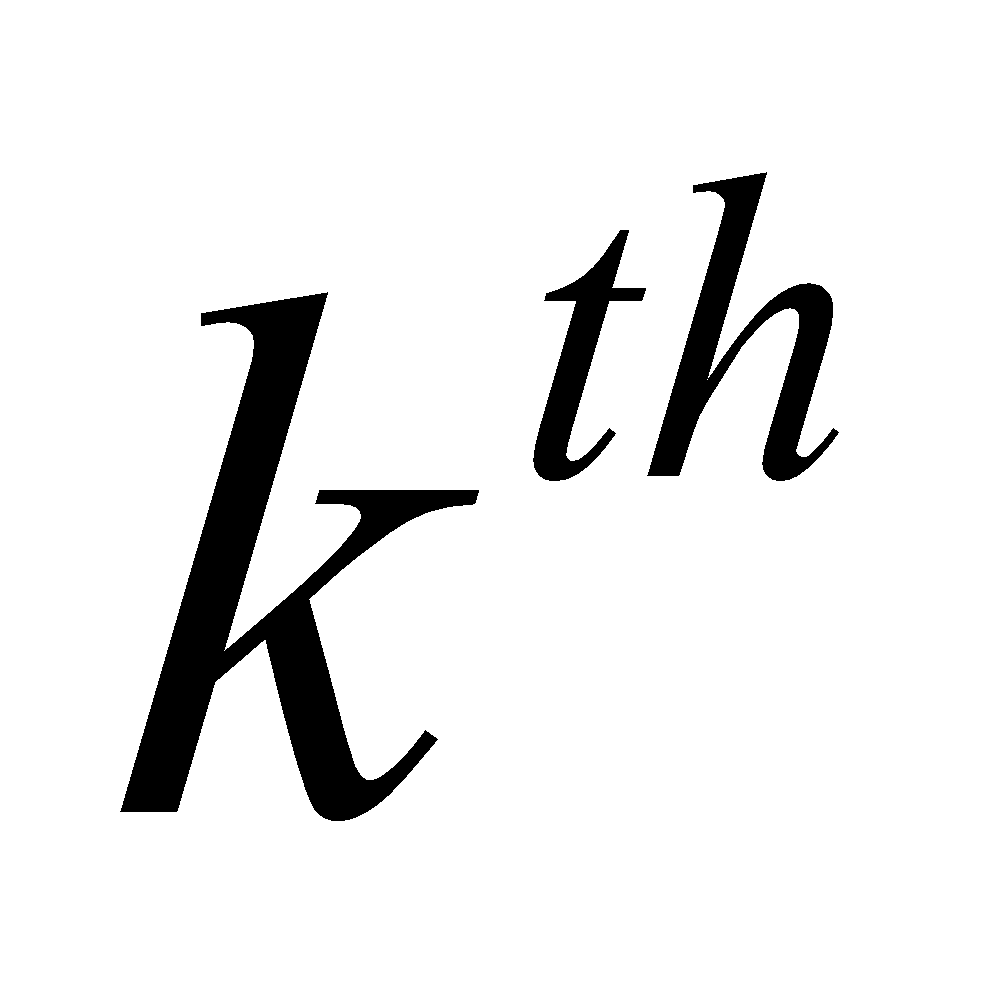
_ group, with corresponding regression coeﬃcient vector _
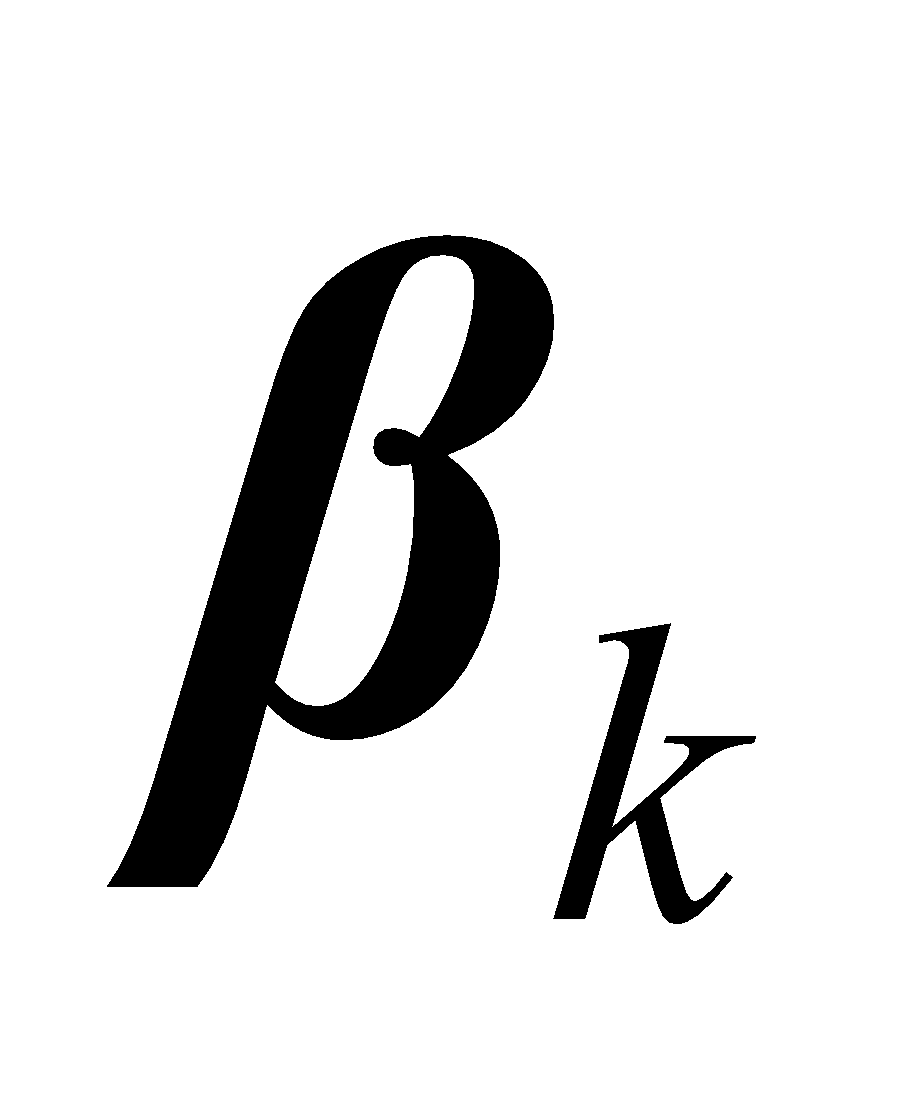
_. The Group Lasso acts like the original Lasso but this time at the group level where depending on value of _
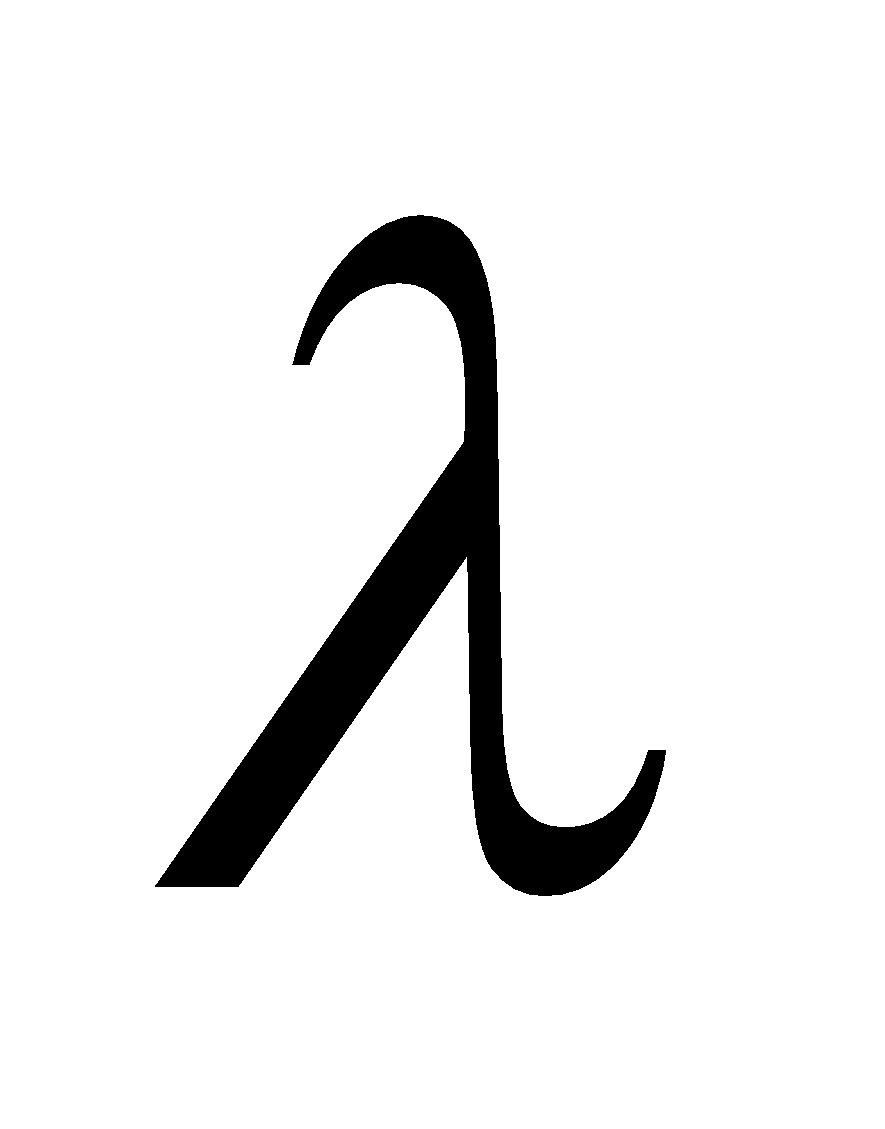
_, an entire group of features may drop out of the model. In fact, if the group sizes are all one, it reduces to the ordinary lasso.

The group lasso does not yield sparsity within a group. That is, if a group of coefficients is non-zero, they are free to have any arbitrary values (similar to the OLS problem). Friedman *et al.* proposed a Sparse Group lasso cost function as follows [(11)](https://paperpile.com/c/s9jEPr/gR1YG):

_
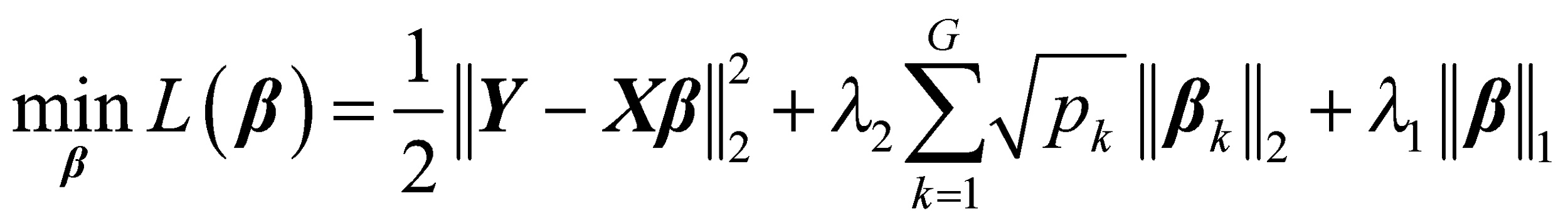
_

_
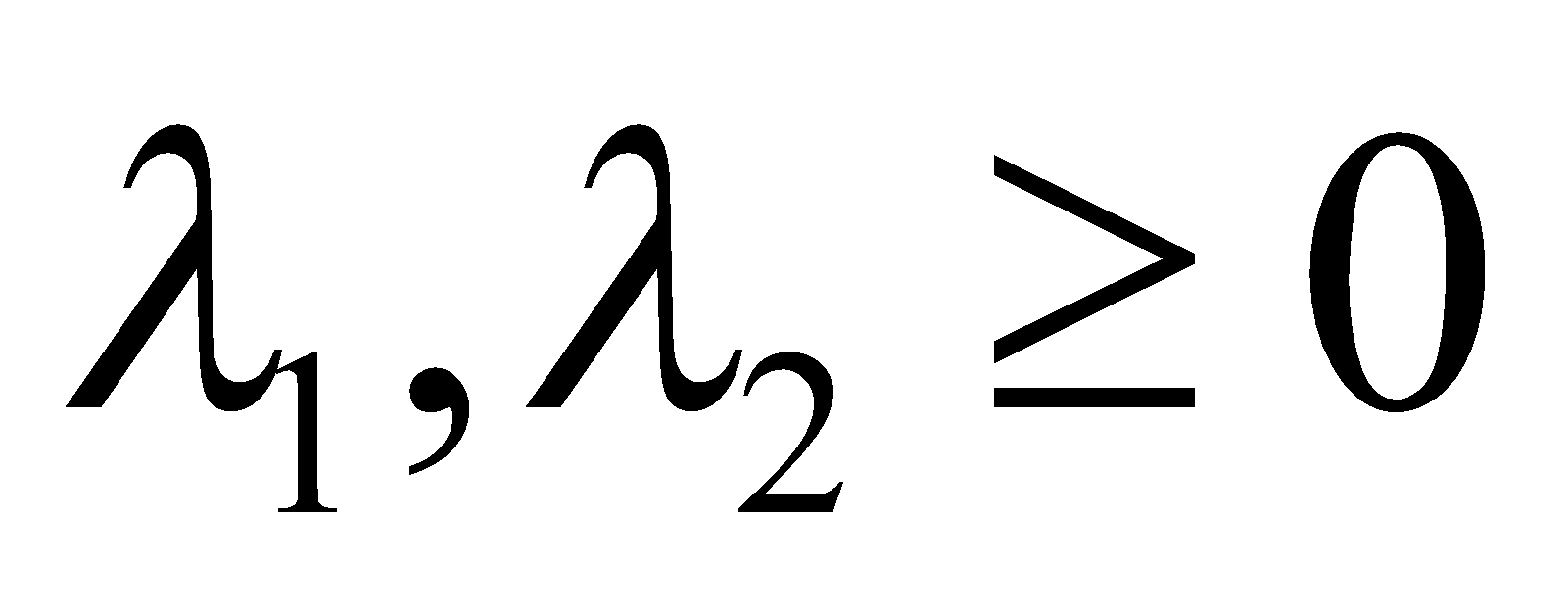
_

It is a more general penalty compared to group lasso that yields sparsity at both groups and individual coefficient levels. For implementation of the Lasso, Group Lasso and Sparse Group Lasso we used SLEP package (v4.1) [(12)](https://paperpile.com/c/s9jEPr/h7Qpe). We further included a wrapper in this package to add support for sample weighting which mitigate unbalanced classes. Additionally a grid search is added for estimating the optimal parameters (i.e. Lambda) using an inner cross-validation. To this end, we swept through 20 Lambdas from interval of 1e-2 to 1 in a logarithmic scale. Similar values are considered for feature regularization in Lasso as well as group regularization in Group Lasso and Sparse Group Lasso. All corresponding implementations are publicly available through github https://github.com/UMCUGenetics/SyNet.

## Topological measures

In our work, a range of graph topological measures are calculated that describe local graph structure around a node or a edge. The degree is defined as the number of edges connected to the node. The shortest path between two nodes is defined as smallest number of edges from one node that need to be traversed to reach the other node. Pairs of nodes that are not connected have a shortest path equal to infinity. Betweenness of a node is the number of times that node resides on a shortest path of any other pair of nodes in the network normalized by total number of possible pairs in the network. Closeness of a node measures the inverse sum of distances from that node to all other nodes in the network. The Jaccard index between two nodes is defined as number of shared neighbors between two nodes normalized by the total number of unique neighbors of those nodes. The clustering coefficient of a node computes the number of links between direct neighbors of that node normalized by total number of possible links between those direct neighbors. The eigenvector centrality of a node is equal to its component of the related eigenvector of the network. The page rank of a node corresponds to probability of a random surfer to visit that particular node [(13)](https://paperpile.com/c/s9jEPr/2Opmg). At each step, this surfer visits a direct neighbor of current node (with probability of $\beta$) or restarts its walk from a randomly chosen node in the network (with probability of $1-\beta$). Pagerank is known to be statistically similar to node degree [(14)](https://paperpile.com/c/s9jEPr/CdPh7).

## Supplementary table 2. Gene enrichment analysis for SyNet clusters

Gene enrichment analysis performed using GSEA [(15)](https://paperpile.com/c/s9jEPr/EHRfH) for genes in each individual cluster of SyNet. Each row refer to a study in which given genes are enriched. Columns respectively represent study name (Gene set name), number of genes in the study (# Genes in Gene Set (K)), a brief explanation of enriched set (description), number of genes in the given SyNet cluster that overlap with get set in the study (# Genes in Overlap (k)), chi-square statistics for significance of enrichment (k/K), p-value of corresponding chi-square statistics (p-value) and corrected q-value after false discovery rate control (FDR q-value).

## S1. Determining the optimal operator for meta-features formation


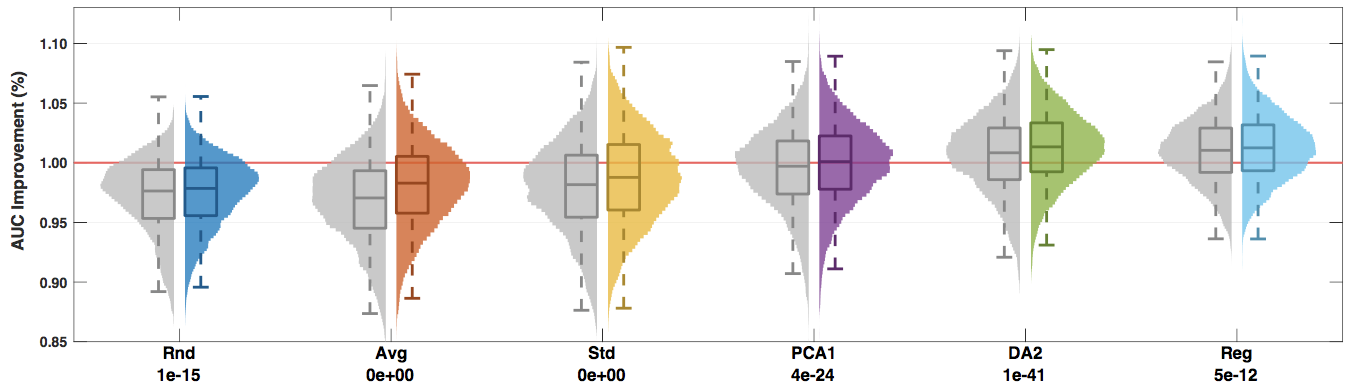


**Figure S1.** Performance gained from meta-genes assembled with different operators. To form a gene set, each gene is selected as “seed” along with its closest 20 neighbor genes according to STRING network. A meta-gene is constructed by averaging the genes (Avg distribution), taking the standard deviation (Std), taking the largest principal component (PCA1), negating the expression of genes that are anti-correlated with outcome before averaging (DA2 [(16)](https://paperpile.com/c/s9jEPr/Y1U0o)) and finally for the Reg meta-gene a linear regression is trained on 50% of patients (randomly selected) and applied to the test set (other 50%) to form the meta-gene. In this figure, the AUC of each meta-gene is compared to the AUC of the best gene in the set (determined in the training set). As a control, we also included a meta-gene where a random gene in the gene set is chosen as the best gene (Rnd). The gray distributions indicate performance gained by a shuffled version of the STRING network (as described in the Methods). The number below each pair of distributions, represents the p-value of a one-sided t-test comparing the distributions. This result shows that the average operator performs on par with the random operator although it is widely used in NOPs. Meanwhile, the DA2 operator, which simply adjust gene directions according to outcome, performs substantially better in improving performance of meta-genes. The top performing operator is regression.

## S2. Poor genes tend to yield more synergy compared to predictive genes

**
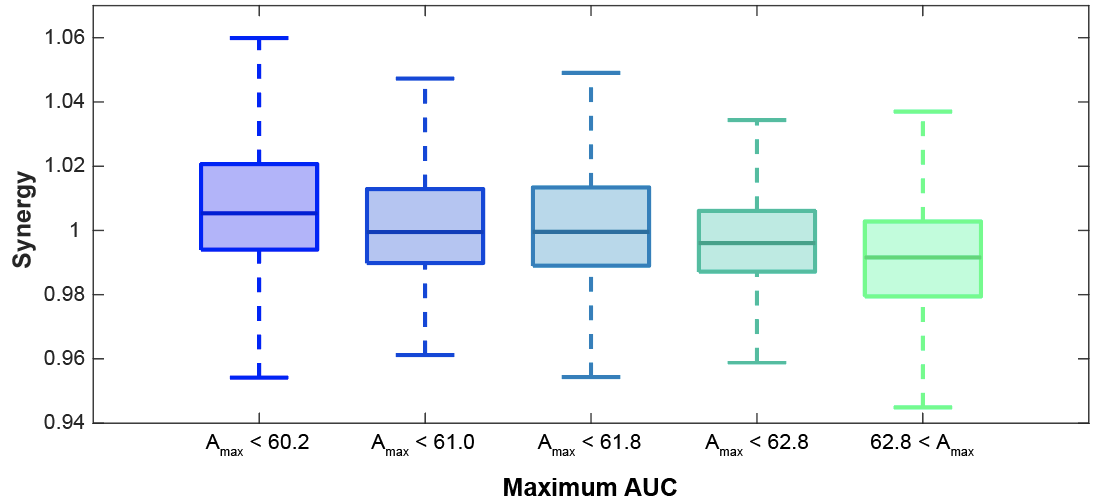
**

**Figure S2.** Poorly performing genes tend to yield more synergy compared to predictive genes. The top 10k pair in SyNet is selected and grouped into 5 non-overlapping bins according to $max(A_{i}, A_{j})$ where $A_{i}$and $A_{j}$ are the AUC of gene i and gene j respectively. For each group, bars represent distribution of synergy ($S_{ij})$. This result show that for higher individual AUCs, synergy is reduced.

## S3. Performance of classical predictors


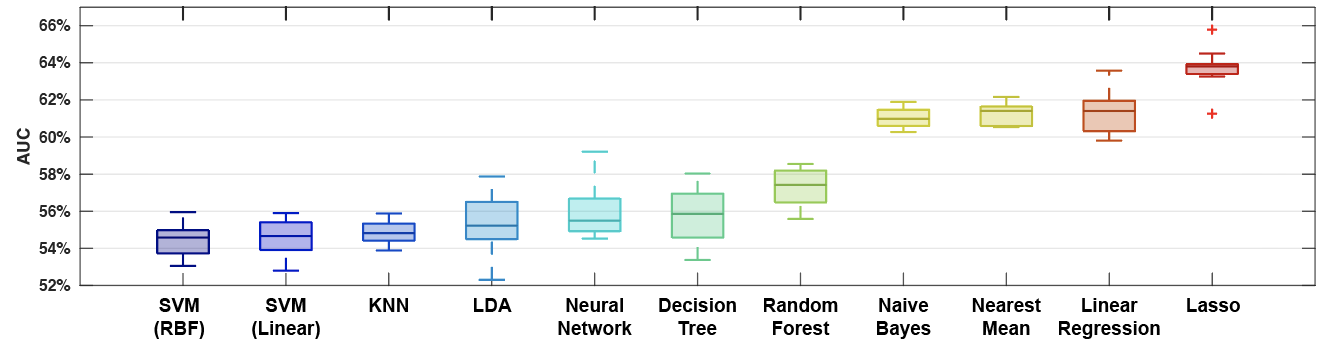


**Figure S3.** Performance of standard classifiers trained using individual features (no network information is utilized). The cross-study validation scheme is employed to evaluate range of linear and nonlinear classifiers including Naive Bayes (NB) , Nearest Mean Classifier (NMC), Linear Discriminant Analysis (LDA), Linear Regression (LR), Lasso, K-Nearest Neighbors (KNN), Support Vector Machine (SVM) using linear and Radial Basis Functions (RBF), Decision-Tree (DT), Random Forest (RF) and Neural Networks (NN) [(17)](https://paperpile.com/c/s9jEPr/y2rb3). The hyperparameters of each model (e.g. the gamma parameter in SVM) is optimized by means of inner fold cross-study evaluations. Samples used to train and validate these classifiers are identical to the samples used for the performance evaluations in the main manuscript. According to these results, linear classifiers offer improved performance compared to non linear (and more complex) classifiers (with exception of LDA). The best performing classifier is the Lasso which regularises gene weights to perform a simultaneous selection and integration of gene expressions. Therefore, performance is improved by marginal increase of complexity.

## S4. Batch effect removal using COMBAT

**
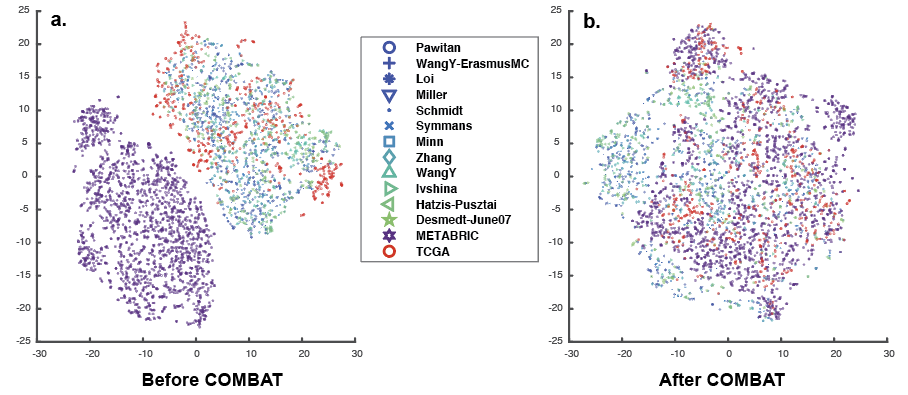
**

**Figure S4.** COMBAT is successfully employed to remove batch effects between studies. **a.** Expression data (n=4109) is first quantile normalized within each study and then visualized using t-SNE (perplexity=20) in two-dimensional space. Based to this visualization, METABRIC data is clearly occupying a different part of gene expression space compared to other studies. **b.** Batch effect removed data after applying COMBAT. According to t-SNE visualization, data from different studies show homogenous patterns in the expression space.

## S5. Cross-validation scheme

**
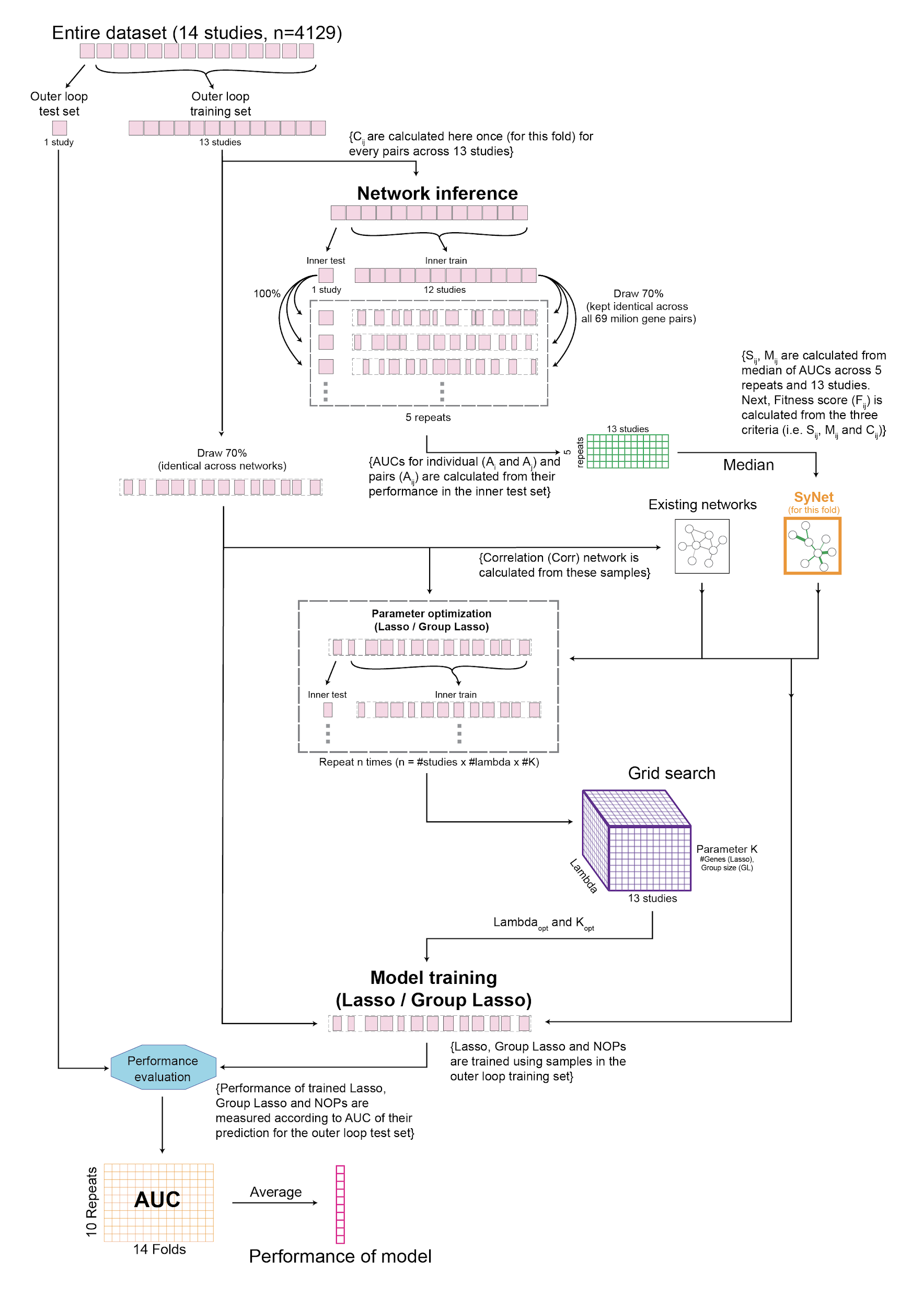
**

**Figure S5.** The utilized cross-validation scheme to investigate performance of NOPs.

## S6. Performance variation in leave one study out cross validation

**
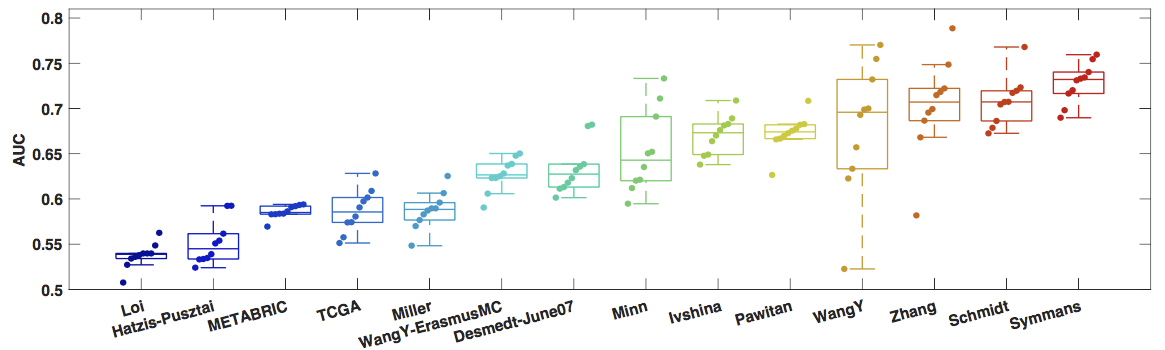
**

**Figure S6.** Performance of Lasso for predicting survival risk of patients is highly variable across studies. To calculate Lasso performance, this classifier is trained using 70% of samples from 13 studies and tested over the entire samples in the left out study. This procedure is repeated 10 times for each study.

## S7. Comparison of performance for networks and groups of identical size


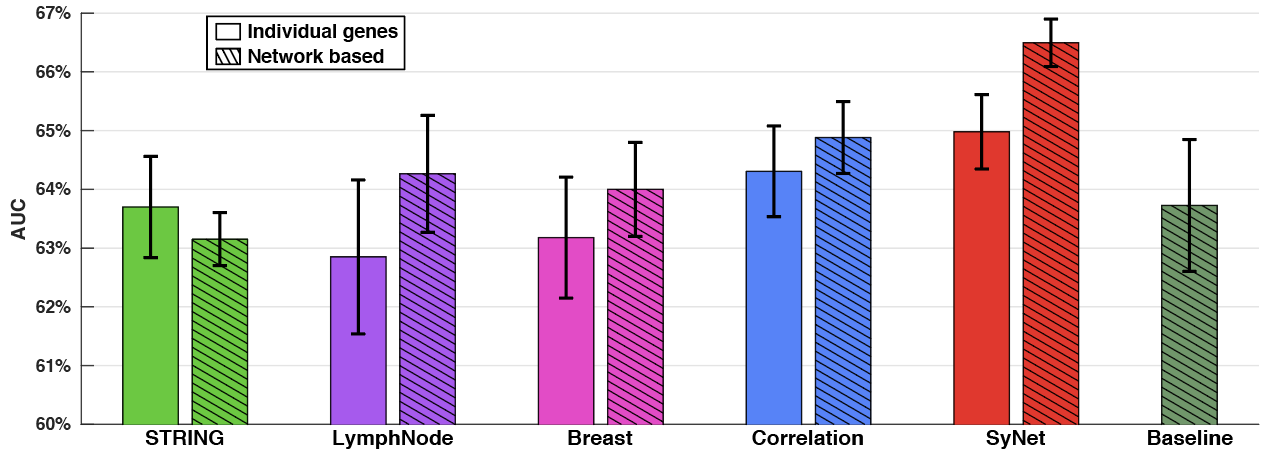


**Figure S7.** Similar trend of performance is observed when number of links in networks are kept identical (n=10000). No grid search for group size is performed in this analysis and group size is kept constant (K=5).

## S8. Sparse group lasso performance compared to group Lasso


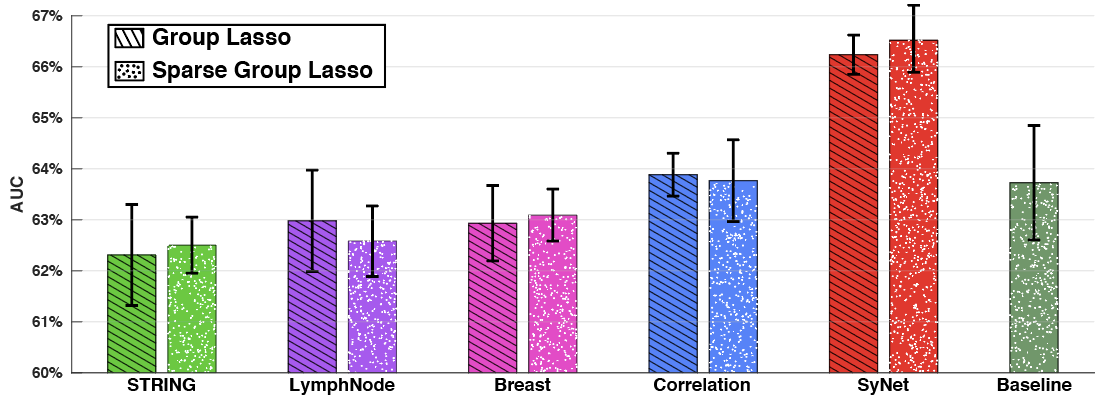


**Figure S8.** While Sparse Group Lasso is computationally more expensive than group Lasso, it does not outperform group Lasso in terms of performance. Identical set of Lambda for both feature level and group level regularization of sparse group lasso is considered. These two parameters are optimized in an inner loop cross-validation fashion as explain in the paper.

## S9. Performance of SyNet does not change if #genes and group size are optimized simultaneously

**
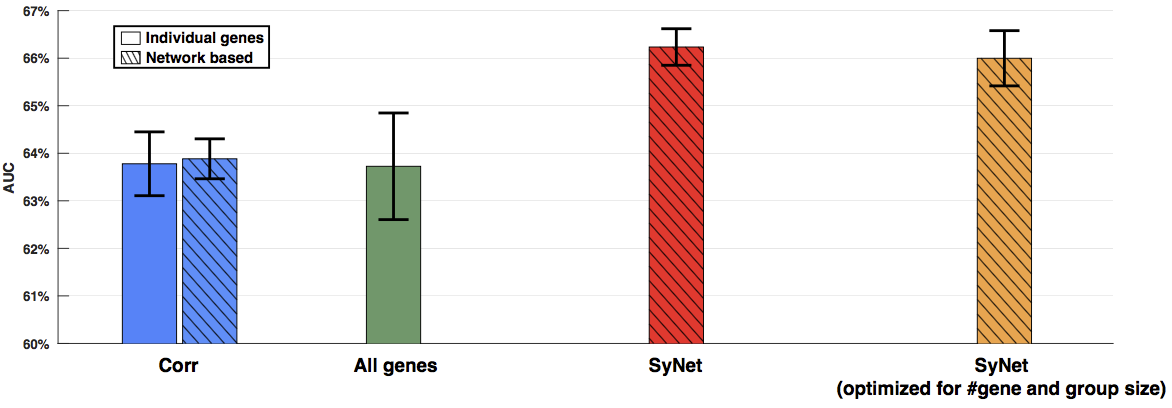
**

**Figure S9.** Performance of SyNet does not change substantially if number of genes and group size are optimized simultaneously. Instead of optimizing group size and number of genes separately (which is done in the paper, red bar), one can optimize these parameters simultaneously (at the cost of computation time). To this end, a grid search is employed to search across set of group sizes (2, 3, 5, 7 and 10) and number of genes (100, 300, 700, 1000, 1500 and 3000) to compare performance of GL in these two settings (i.e. separate vs. simultaneous optimization). The results indicates that performance of this concurrently optimized model (orange bar) does not change substantially compared to a case when these parameters (group size and number of genes) are optimized separately.

## S10. Similarity between biological networks and SyNet without the correlation criterion


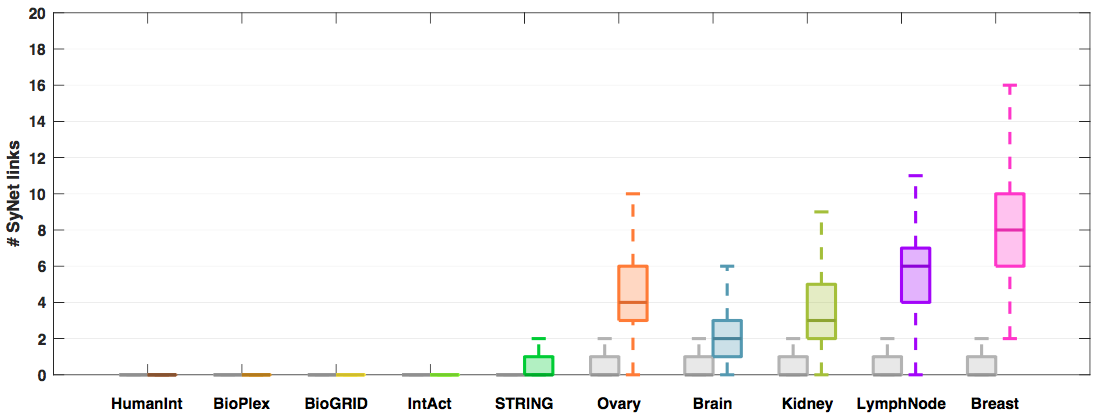


**Figure S10.** Overlap of SyNet links with existing biological networks is not purely driven by correlation criterion. To investigate this, Fitness $F_{ij}$ of all pairs are calculated only using synergy ($S_{ij}$) and average AUC ($M_{ij}$) and ignoring correlation component (i.e. $F_{ij}= -\sqrt{{(1-\overline{S_{ij}})}^{2}+(1-\overline{M_{ij}})^{2}}$). Similar to the analysis presented in the main paper, the existence of top SyNet pairs (n=3544, according to the new Fitness) in existing biological networks is assessed by randomly sampling equal number of links (n=3544) in the biological network. The frequency of observing overlapping links are depicted as boxplots. Gray box plots indicate the same analysis performed on the shuffled version of the biological networks.

**S11. Precision recall curves for overlap between SyNet and existing networks**

| **a.**  **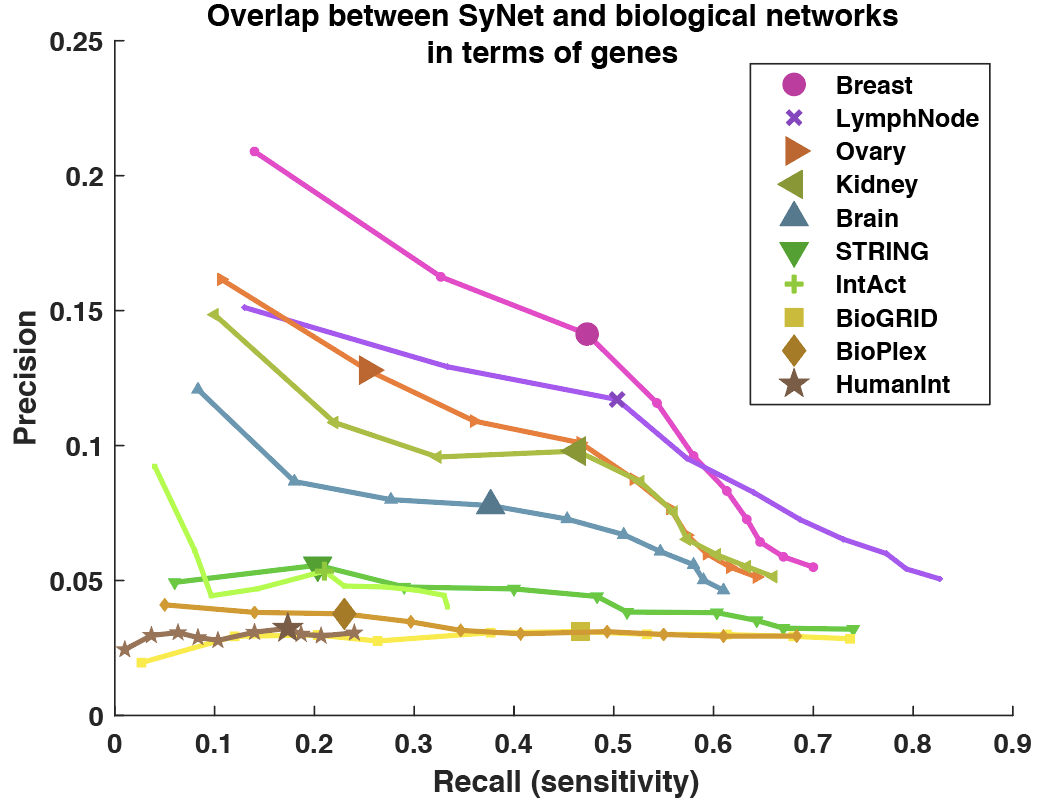** | **b.**  **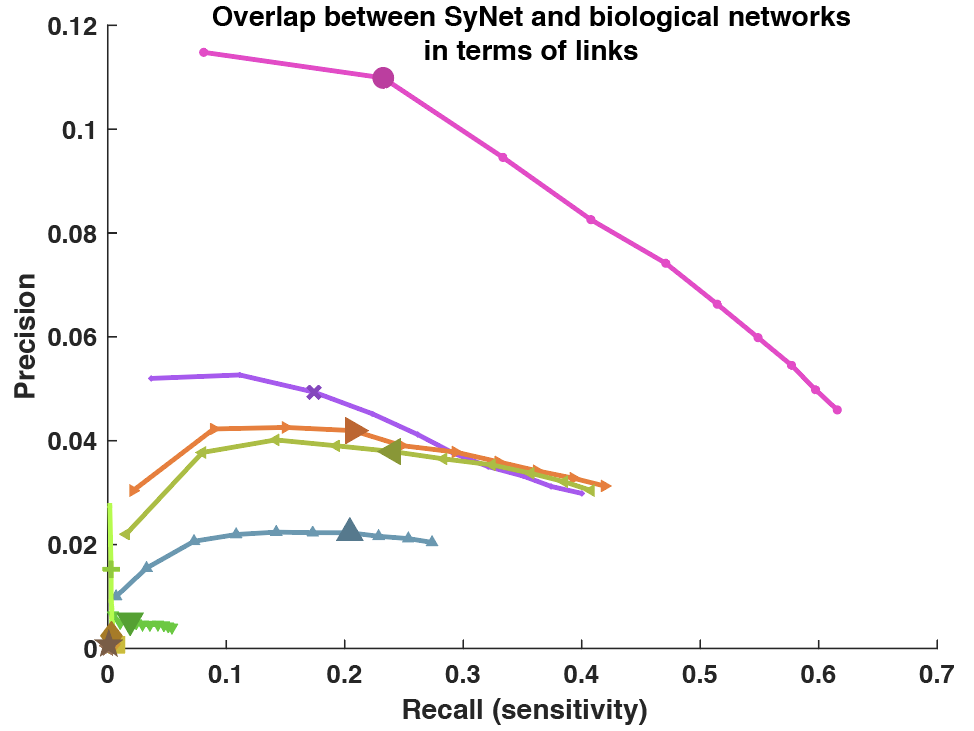** |
| --- | --- |

**Figure S11.** Precision recall curves for overlap between SyNet and existing networks. Existing biological networks miss many (**a.**) genes and (**b.)** links necessary for outcome prediction. The curves demonstrate the degree of similarity between SyNet genes and links that are also present in biological networks across set of thresholds (5%-100% of total genes/links with steps of 5%). Large markers indicate the threshold with maximum F1-score (computed from precision and recall at each threshold) across considered thresholds.

**S12. Performance of Corr network compared to shuffled version**


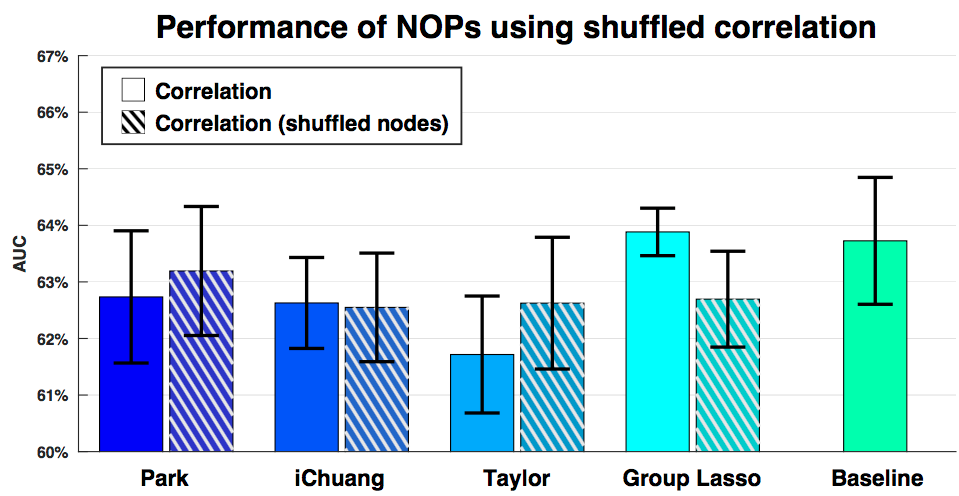


**Figure S12.** Performance of correlation network do not show deterioration in existing NOPs but it does when group lasso used. This indicates that existing NOPs do not effectively incorporate interactions in the given network.

**S13. Performance of top outcome predictors with limited samples (subsampling analysis)**

We focused only on the top three predictive networks (i.e. STRING, Correlation and SyNet) and trained a Group Lasso model using an identical set of (training and test) samples as used in the main manuscript for each network. Additionally, we evaluated performance of the baseline model (i.e. Lasso using all genes available in our collected dataset, n=11748). Results of this experiment are represented in **Figure S13**.

**
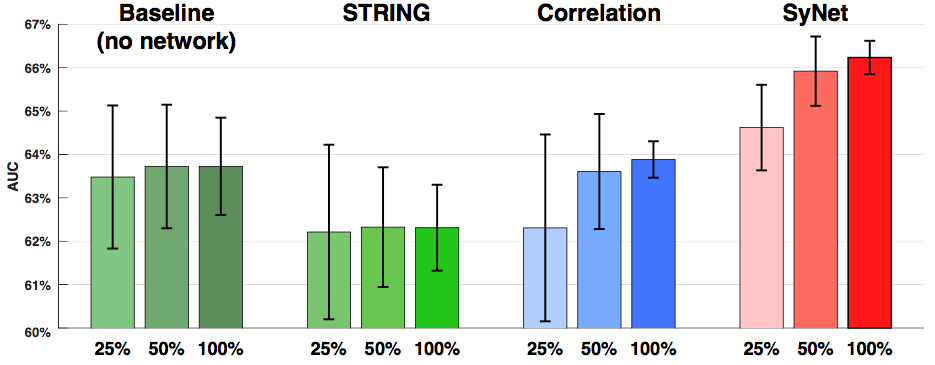
**

**Figure S13.** Performance of top three predictive networks (as well as the baseline model) when trained using 25% or 50% of samples available in our dataset. SyNet outperforms other models even though it uses only 25% of samples to infer its synergistic network.

As expected, our assessment shows reduced classification performance for all models. This reduction is most severe for models that use the training data to infer corresponding network (i.e. Correlation and SyNet). Interestingly, we found that even using 25% of training data, a Group Lasso model guided by SyNet performs better than a model that is guided by Correlation network (the second-best performing network). This shows that, even with a limited number of samples, data-driven gene networks can guide training of outcome predictors.

**S14. Performance of hub genes in SyNet**

We selected genes in SyNet with at least 5 neighbors (i.e. degree >=5) and trained Lasso as well as Group Lasso across 14 folds and 10 repeats using identical samples as the analyses in the main manuscript. The median number of genes used in each fold was 175, meaning that nearly half of genes in the original SyNet were given to these “hub-based models”. Results of this experiment are visualized in **Figure S14**.


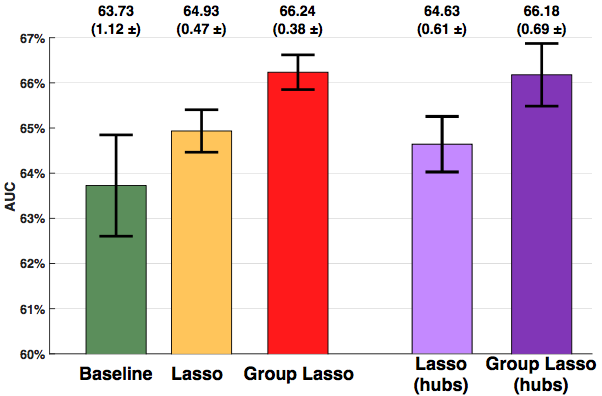


**Figure S14.** Performance of Lasso and Group lasso when only hub (degree >= 5) genes are used. The average and standard deviation of the performance for each model across 14 studies and 10 cross-validation repeats are represented by bars and error bars respectively. These values (i.e. mean and standard deviation) are also represented by numbers above each bar respectively.

Our results show that both Lasso and Group Lasso provide similar performance if they are limited specifically to hub (i.e. degree >=5) genes. However, these models exhibit a slightly larger standard deviation of the performance indicative of a reduced stability of the performance. Therefore, we argue that although “core” genes are important in performance of outcome predictors, proper expression integration of core genes and their (synergistic) neighbors can plays a crucial role in performance and stability of the outcome predictors.

**S15. Performance of merged networks**

To investigate whether a combination of networks would provide a better performance compared to individual networks, we merged 100,000 top links from the top performing networks including STRING, Breast and LymphNode (in total of 202237 links after removal of duplicates) and trained Lasso and Group Lasso to predict survival of unseen patients across independent studies. For this assessment, we utilized an identical set of training and test samples that are also used in the main manuscript. **Figure S15** represents the result of this experiment. According to these results, combining links from multiple network does not improve performance of classical or network-based outcome predictors. This could be result of excessive number of links that are used from these networks (i.e. curse of dimensionality), or lack of confidence for the included links (as threshold is reduced to include 100k links) which further masks the predictive information in the utilized links.


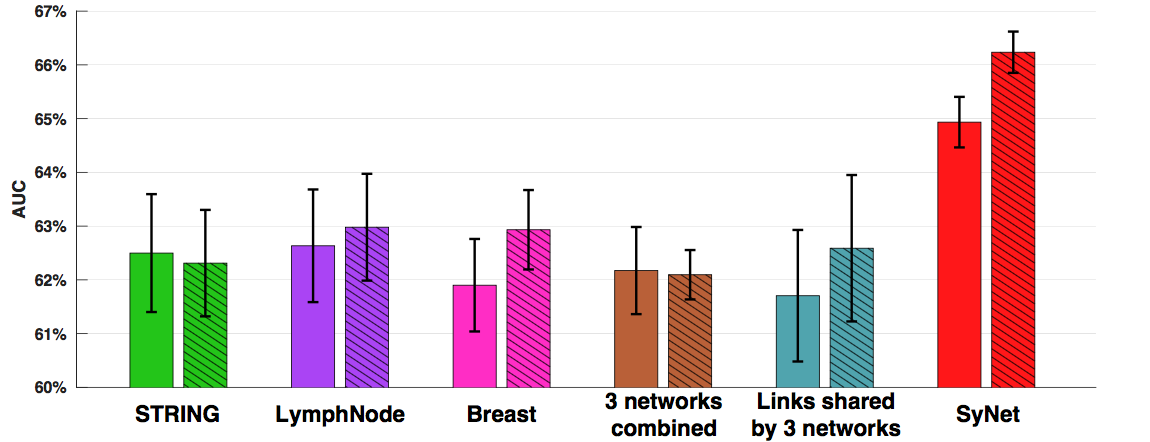


**Figure S15.** Performance of top three networks compared to a case when top 100k links from each network are combined into a single aggregated network (#link=~202k) as well as when top shared links in these networks are used in a cross-study validation procedure. SyNet substantially outperforms these networks although it contains only 300 genes and ~3000 links.

To investigate whether using links with high confidence helps to boost performance of these models, we formed a new network using top one million links in STRING, LymphNode and Breast (in total 3 million links) and selected pairs that are present in all three networks (n=77961 links connecting n=5986 genes). Next, we trained Lasso and Group Lasso using the identical settings as the main manuscript (14 folds, 10 repeats). Corresponding model performances are depicted in **Figure S15** (dark blue bars). Our analysis shows that using links that are shared by multiple networks have a modest positive impact on Group Lasso’s performance. However, reduction of Lasso performance (which only uses genes in this network) hints to lower performance of (individual) genes included in the newly formed network. This could be explained by the fact that intersecting genes from diverse networks would result in selection of broadly active genes that may have a lower specificity to the tissue or even more importantly the disease of interest. In agreement with our conclusion in this paper, we argue that a disease specific measure of selection should be implemented in NOPs to ensure that genes and more crucially their corresponding links contain predictive information that could be extracted by the final classifier.

**S16. Performance of models under study do not change with more populated networks**

In the analyses throughout our manuscript, we limited networks under study to have maximum of 50k links to maintain a reasonable network size (in terms of number of links) which results in reducing computational burden. As gene groups in our analyses are formed according to the top weighted neighbors for each gene, reducing confidence threshold (which results in more links in each network) may have little to no influence in the final set of gene groups formed. To demonstrate this effect, we increased number of links for the top three networks (i.e. STRING, LymphNode and Breast) from 50k to 100k, 250k and 500k and trained Lasso and Group Lasso using identical settings as the analyses in the original manuscript (i.e. 14 folds and 10 repeats). Result of this experiment is represented in **Figure S16**. This result demonstrates the minor influence of this threshold on the performance of models under study.


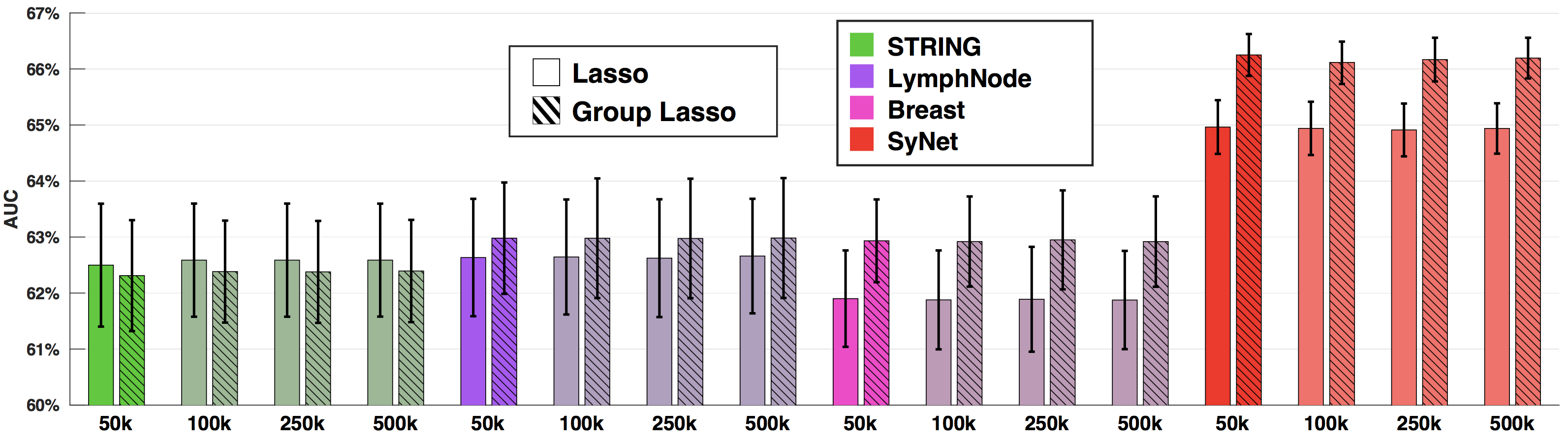


**Figure S16.** Performance of the models under study show minor changes when confidence threshold is reduced to include more links in the network. Mean and standard deviation of performances across 10 repeats are denoted by bars and error bars respectively. Numbers below each bar represent number of links used.

**S17. Trend of overlap between networks and SyNet do not change the #top links selected is varied**

To investigate whether network size threshold has an impact in our conclusions, we performed an overlap analysis for all 10 networks under study (i.e. BioGRID, Breast, Lymph node, etc.) using four smaller and larger thresholds (namely: 10k, 100k, 250k and 1000k links). To form a binary network for each threshold X, we utilized top X number of links in each network. Next, we randomly selected 3544 links (equal to number of links in SyNet) and asked how many of these selected links are also present in SyNet. This procedure is repeated 1000 times to produce an “observed” number of SyNet links in the networks under study. To estimate an expected distribution of SyNet links, we repeated the same experiment while nodes in each thresholded network were randomly swapped with other nodes in the network in each selected round. A summary result of this analysis is represented in **Figure S17**. As expected, the distribution of observed vs. expected number of SyNet links become more similar when network size increases (due to presence of more irrelevant and low confidence links). According to this result, changing network size threshold has a minor effect in the observed trend in the main paper. Therefore, in corroboration with our argument in the main paper, we conclude that more related tissue-specific networks (i.e. Breast and Lymph node) show larger overlap with SyNet compared to other networks (i.e. other tissue specific or generic networks).

**Figure S17.** Overlaps between phenotype-naive networks and SyNet show a similar trend across smaller or larger network sizes. Overlap analysis between links for network under study when limited to **a.** 10k links, **b.** 50k links, **c.** 100k links, **d.** 250k links and **e.** 1000k links. As expected, observed vs. expected number of SyNet links are more similar for larger network sizes.

**S18. Phenotype-specific networks provide superior performance compared to Subtype-specific network of genes in outcome prediction**

We collected three subtype specific networks (i.e. Basal-A, Basal-B and Luminal) inferred by Zaman et al (18). These networks are collected from bri.nrc.ca/wang and combined to form a Subtype Specific Breast Cancer network (SSBC). We utilized all genes and links in SSBC network to train and test both Lasso and Group Lasso classifiers across 14 folds and 10 repeats using identical (train/test) samples as the main manuscript. The result of this experiment is represented in **Figure S18**.


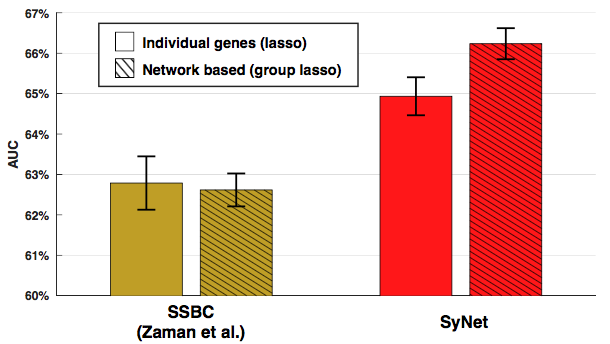


**Figure S18.** Performance of Subtype Specific Breast Cancer (SSBC) network compared to SyNet.

# References

[1. Goldman,M., Craft,B., Zhu,J., Swatloski,T., Cline,M. and Haussler,D. (2016) Abstract 5270: The UCSC Xena system for integrating and visualizing functional genomics. *Cancer Res.*, **76**, 5270–5270.](http://paperpile.com/b/s9jEPr/YQfjL)

[2. Rolland,T., Taşan,M., Charloteaux,B., Pevzner,S.J., Zhong,Q., Sahni,N., Yi,S., Lemmens,I., Fontanillo,C., Mosca,R., *et al.* (2014) A proteome-scale map of the human interactome network. *Cell*, **159**, 1212–1226.](http://paperpile.com/b/s9jEPr/0xGA0)

[3. Huttlin,E.L., Bruckner,R.J., Paulo,J.A., Cannon,J.R., Ting,L., Baltier,K., Colby,G., Gebreab,F., Gygi,M.P., Parzen,H., *et al.* (2017) Architecture of the human interactome defines protein communities and disease networks. *Nature*, **545**, 505–509.](http://paperpile.com/b/s9jEPr/ipl63)

[4. Chatr-Aryamontri,A., Oughtred,R., Boucher,L., Rust,J., Chang,C., Kolas,N.K., O’Donnell,L., Oster,S., Theesfeld,C., Sellam,A., *et al.* (2017) The BioGRID interaction database: 2017 update. *Nucleic Acids Res.*, **45**, D369–D379.](http://paperpile.com/b/s9jEPr/ujy0k)

[5. Szklarczyk,D., Morris,J.H., Cook,H., Kuhn,M., Wyder,S., Simonovic,M., Santos,A., Doncheva,N.T., Roth,A., Bork,P., *et al.* (2016) The STRING database in 2017: quality-controlled protein–protein association networks, made broadly accessible. *Nucleic Acids Res.*, **45**, D362–D368.](http://paperpile.com/b/s9jEPr/t4Qxz)

[6. Greene,C.S., Krishnan,A., Wong,A.K., Ricciotti,E., Zelaya,R.A., Himmelstein,D.S., Zhang,R., Hartmann,B.M., Zaslavsky,E., Sealfon,S.C., *et al.* (2015) Understanding multicellular function and disease with human tissue-specific networks. *Nat. Genet.*, **47**, 569–576.](http://paperpile.com/b/s9jEPr/mn6Bn)

[7. Kinsella,R.J., Kähäri,A., Haider,S., Zamora,J., Proctor,G., Spudich,G., Almeida-King,J., Staines,D., Derwent,P., Kerhornou,A., *et al.* (2011) Ensembl BioMarts: a hub for data retrieval across taxonomic space. *Database* , **2011**, bar030.](http://paperpile.com/b/s9jEPr/upBDE)

[8. Tibshirani,R. (2011) Regression shrinkage and selection via the lasso: a retrospective. *J. R. Stat. Soc. Series B Stat. Methodol.*, **73**, 273–282.](http://paperpile.com/b/s9jEPr/oLhT)

[9. Tibshirani,R. (1996) Regression Shrinkage and Selection via the Lasso. *J. R. Stat. Soc. Series B Stat. Methodol.*, **58**, 267–288.](http://paperpile.com/b/s9jEPr/5bQ8)

[10. Yuan,M. and Lin,Y. (2006) Model selection and estimation in regression with grouped variables. *J. R. Stat. Soc. Series B Stat. Methodol.*, **68**, 49–67.](http://paperpile.com/b/s9jEPr/I1BUy)

[11. Friedman,J., Hastie,T. and Tibshirani,R. (2010) A note on the group lasso and a sparse group lasso.](http://paperpile.com/b/s9jEPr/gR1YG)

[12. Liu,J., Ji,S., Ye,J. and Others (2009) SLEP: Sparse learning with efficient projections. *Arizona State University*, **6**, 7.](http://paperpile.com/b/s9jEPr/h7Qpe)

[13. Gross,J.L., Yellen,J. and Zhang,P. (2013) Handbook of Graph Theory, Second Edition CRC Press.](http://paperpile.com/b/s9jEPr/2Opmg)

[14. Perra,N. and Fortunato,S. (2008) Spectral centrality measures in complex networks. *Phys. Rev. E Stat. Nonlin. Soft Matter Phys.*, **78**, 036107.](http://paperpile.com/b/s9jEPr/CdPh7)

[15. Subramanian,A., Tamayo,P., Mootha,V.K., Mukherjee,S., Ebert,B.L., Gillette,M.A., Paulovich,A., Pomeroy,S.L., Golub,T.R., Lander,E.S., *et al.* (2005) Gene set enrichment analysis: a knowledge-based approach for interpreting genome-wide expression profiles. *Proc. Natl. Acad. Sci. U. S. A.*, **102**, 15545–15550.](http://paperpile.com/b/s9jEPr/EHRfH)

[16. Allahyar,A. and de Ridder,J. (2015) FERAL: network-based classifier with application to breast cancer outcome prediction. *Bioinformatics*, **31**, i311–9.](http://paperpile.com/b/s9jEPr/Y1U0o)

[17. Mitchell,T.M. (1997) Machine Learning McGraw-Hill, Inc.](http://paperpile.com/b/s9jEPr/y2rb3)

18. Zaman, N., Li, L., Jaramillo, M. L., Sun, Z., Tibiche, C., Banville, M., ... & O’Connor-McCourt, M. (2013). Signaling network assessment of mutations and copy number variations predict breast cancer subtype-specific drug targets. Cell reports, 5(1), 216-223.
